# Mechanistic Insights into the Regioselective (3 + 2) Cycloaddition of Unsymmetrical Cyclopropenones with Elemental Sulfur: Experimental and Computational Studies

**DOI:** 10.1021/acs.joc.5c01955

**Published:** 2026-01-14

**Authors:** Pablo Rivero, Gonzalo D. Nuñez, Eric Miró, Mario Villares, Jorge J. Carbó, Sergio Castillón, Yolanda Díaz, Maria Besora, María Isabel Matheu

**Affiliations:** † Departament de Química Analítica i Química Orgànica, 16777 Universitat Rovira i Virgili, C/Marcel·lí Domingo 1, 43007 Tarragona, Spain; ‡ Departament de Química Física i Inorgànica, Facultat de Química, 16777 Universitat Rovira i Virgili, C/Marcel·lí Domingo 1, 43007 Tarragona, Spain

## Abstract

This study investigates the (3 + 2) cycloaddition of substituted cyclopropenones with elemental sulfur. Combined experimental and computational results highlight the involvement of reactive sulfur species, such as those generated from KF or sulfide impurities. Oxygen exerts minimal influence, whereas oxidants such as BQ or *m*-CPBA completely suppress the reaction. DFT calculations challenge conventional S_8_-based mechanisms, pointing to an energetically costly process from inactivated S_8_. Our results support that the cyclic S_8_ molecules of elemental sulfur are inactive toward cyclopropenone reagents. The true reactive species are polysulfide anions, which can be present in different forms (FS_*n*_^–^, HS_*n*_^–^, or S_*n*_^2–^) depending on the additive type and purity of the sulfur source. These species undergo conjugate addition to the least substituted carbon of the cyclopropenone C=C bond, ultimately affording a five-membered heterocycle—1,2-dithiol-3-one—as a single regioisomer, through a ketenethialdehyde and thiet-2-one sequence.

## Graphical abstract

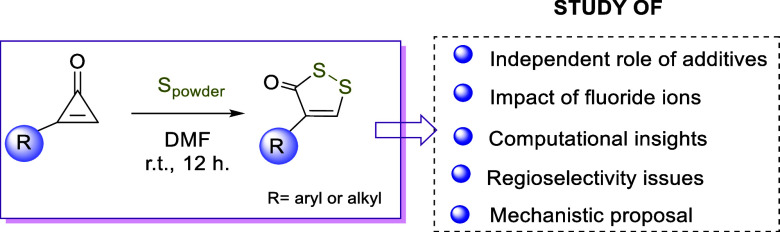

## Introduction

The versatile reactivity of elemental sulfur offers opportunities to design efficient and sustainable transformations in organic synthesis.^1^ Elemental sulfur is a cheap, odorless, low-toxic, nonhygroscopic, and stable sulfur source under ambient conditions. External reagents such as acids,^2^ bases,^3^ metal complexes,^4^ nucleophiles (e.g., ammonia, cyanides, isocyanides, amines, or triphenylphosphine),^5^ or the use of photocatalytic conditions,^6^ among others, can activate elemental sulfur in mild conditions via homolytic and/or heterolytic pathways, thus expanding its reactivity.

Given its user-friendly nature as a synthetic tool, elemental sulfur undoubtedly stands as the best source of sulfur atoms in the synthesis of sulfur-containing heterocycles.^1b,1c,1e^ In this field, and in the context of our work related to ceramides containing a cyclopropenone ring as a rigid scaffold,^7^ we became interested in the work of Wu et al.^8^ reporting the synthesis of 1,2-dithiol-3-ones from cyclopropenone derivatives via (3 + 2) cycloaddition by using elemental sulfur. The described protocol exhibited high efficiency, atom economy, gram-scale capacity, and a wide scope. Thus, symmetrical diaryl or dialkyl cyclopropenones furnished the corresponding dithia heterocycles (Scheme 1, R = aryl or alkyl) in high yields under the optimized conditions (KF, dimethylformamide (DMF), air atmosphere, r.t., 12 h).

**Scheme 1:**
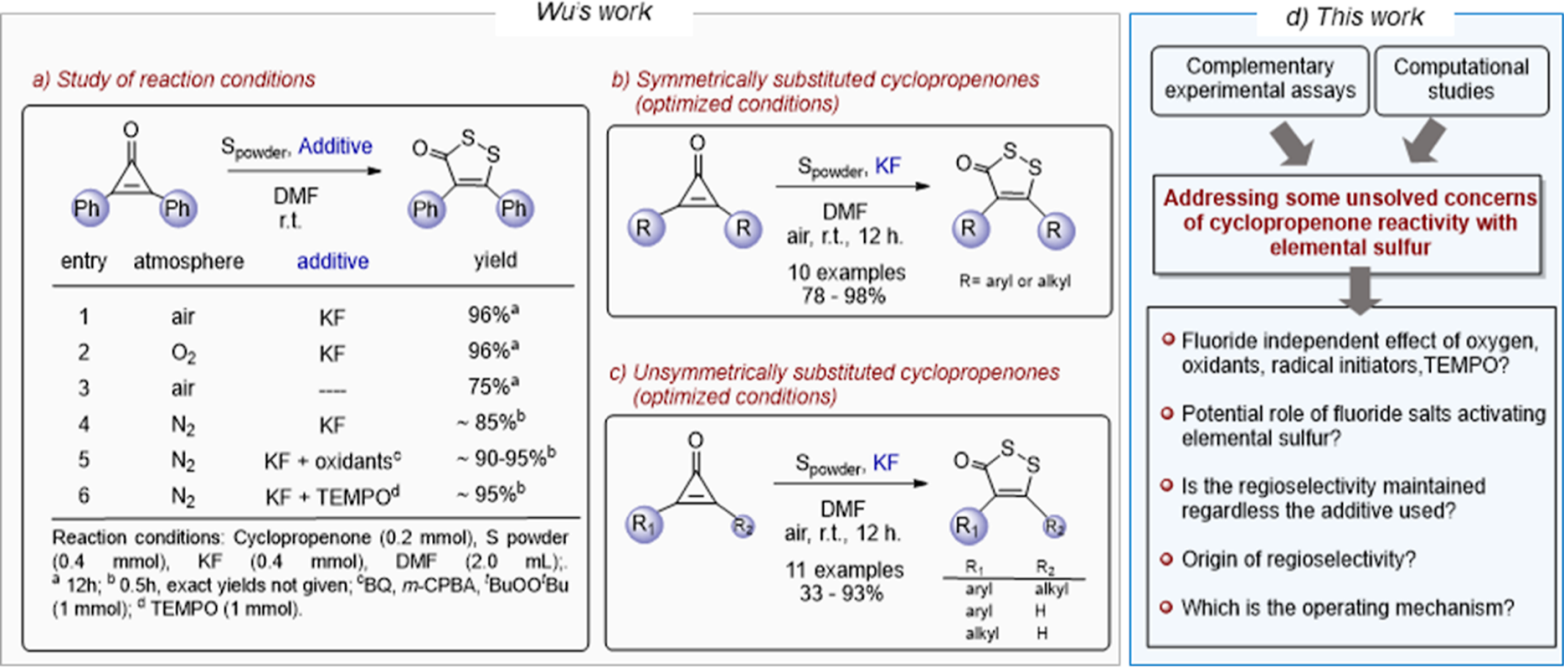
Wu’s Work:^8^ (a) Study of Reaction Conditions and Additives on the Reaction between Diphenylcyclopropenone and Elemental Sulfur; (b) Summary of Results Obtained from Symmetrically Substituted and (c) from Unsymmetrically Substituted Cyclopropenones under the Optimized Conditions; and (d) Our Work: Addressing Unsolved Concerns of This Reactivity

Comparable yields have been obtained using various fluoride sources, including NaF and CsF.^8^ Additionally, the use of catalytic TBAF, with elemental sulfur in tetrahydrofuran, has been reported to provide similar efficiency while allowing for a simpler workup.^9^

Exploration of the reaction conditions described by Wu et al.^8^ from the symmetrically substituted diphenylcyclopropenone (Scheme 1a), shows that although the optimized protocol involves the use of KF in air (Scheme 1a, entry 1) or an oxygen atmosphere (Scheme 1a, entry 2), the reaction also proceeds well without fluoride salts in air (Scheme 1a, entry 3) or with KF under a nitrogen atmosphere (Scheme 1a, entry 4), albeit with a slightly lower yield. Likewise, the use of additives such as 1,4-benzoquinone (BQ), *m*-CPBA, di-*tert*-butyl peroxide (^*t*^BuOO^*t*^Bu) (Scheme 1a, entry 5), or TEMPO (Scheme 1a, entry 6) under N_2_ atmosphere in the presence of KF has been described to promote the formation of the product, yielding similar results to those obtained under an air atmosphere.

The optimized conditions were then applied to symmetrically (Scheme 1b) and unsymmetrically substituted cyclopropenones (Scheme 1c), notably affording a single regioisomer in the latter case, albeit with a broader range of yields (33–93%) compared to those obtained with symmetrical substrates (78–98%). These experimental results raise new and important questions about this reaction, such as the fluoride-independent effect of air (oxygen), oxidants, or TEMPO, an aspect unexplored in Wu’s original study and not easily explained.

Additionally, no experimental validation of the observed regioselectivity has been reported in the absence of fluoride ions, as optimized studies were limited to the symmetric diphenylcyclopropenone (Scheme 1a).

Additionally, the different mechanistic proposals found in the literature have not been supported by computational studies and do not clearly explain the high regioselectivity of the process. In this sense, the mechanism proposed by Wu and co-workers^8^ involved the addition of **S**_**8**_ to the cyclopropenone carbonyl group (Scheme 2a, intermediate **I**), followed by the release of S_6_, furnishing **II**, which would then undergo a tandem ring opening/cyclization sequence, ultimately yielding the heterocyclic product. An alternative mechanism proposed by Klimova^10^ (Scheme 2b) involves the initial nucleophilic attack of molecular sulfur to an olefinic carbon atom of the three-membered ring, to afford intermediate **IV**, followed by a ring opening leading to intermediate **V** and a final ring closure process. The only mechanism that accounts for the potential role of fluoride salts in activating elemental sulfur is that proposed by Mlostoń^9^ (Scheme 2c). It suggests a nucleophilic attack by the activated sulfur species on the carbonyl carbon, leading to an anionic intermediate **VI** in equilibrium with the open-chain intermediate **VII**, which ultimately undergoes cyclization via displacement of the –S_6_F group.

**Scheme 2:**
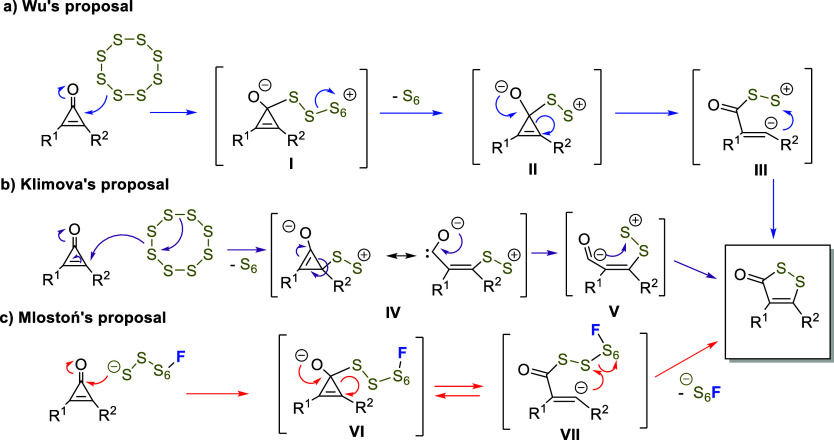
Reproduction of the Proposed Mechanisms for the (3 + 2) Cycloaddition of Cyclopropenone Derivatives and Elemental Sulfur: (a) Wu’s Proposal,^8^ (b) One of Klimova’s Proposals (Another from Intermediate II Has Been Omitted for Clarity),^10^ and (c) Mlostoń’s Proposal^9^

The present study (Scheme 1d) aims to address the aforementioned issues related to the effect of additives (Scheme 1a) and the observed complete regioselectivity (Scheme 1c) by conducting experimental assays complementary to those reported by Wu, as well as performing a computational mechanistic study of the reaction between unsymmetrically substituted cyclopropenones and elemental sulfur. This approach is expected to provide deeper insights into the behavior and underlying mechanism of this (3 + 2) cycloaddition reaction.

## Results and Discussion

### Individual Effect of Additives

The reactivity of two unsymmetrically substituted cyclopropenones, 2-tridecylcycloprop-2-ene-1-one (**1**), and 2-phenylcycloprop-2-ene-1-one (**2**), toward the (3 + 2) cycloaddition reaction was investigated under a variety of conditions to assess the individual impact of selected additives on the outcome of the reaction as well as to experimentally validate the regioselectivity of the (3 + 2) cycloaddition. Reactions from **1** and **2** proceeded under a nitrogen atmosphere without any additives (Table 1, entry 1), affording the expected products **3** and **4** as the sole regioisomers,^11^ in 59% and 29% yield, respectively. Based on the yields, the process conducted in air (Table 1, entry 2) proved to be nearly as efficient as under a nitrogen atmosphere.

**Table 1:**
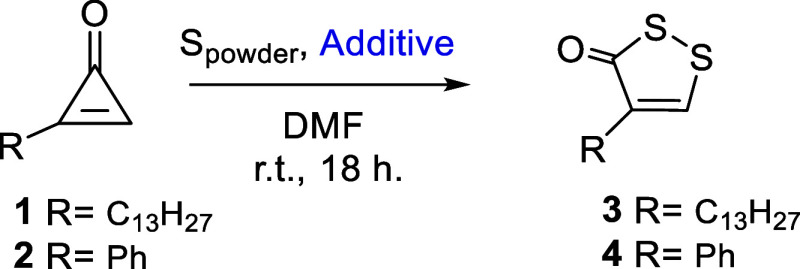
Study of Reaction Conditions in the Reactivity of Unsymmetrically Substituted Cyclopropenones with Sulfur^f^

			yield^a^	
entry	atmosphere	additive	**3**	**4**
1	N_2_	---	59%	29%
2	air	---	62%	30%
3	N_2_	KF^b^	77%^c^	37%
4	air	KF^b^	68%	39%
5	N_2_	TEMPO^d^	72%	32%
6	air	TEMPO^d^	79%	40%
7	N_2_	^*t*^BuOO^*t*^Bu^d^	52%	31%
8	air	^*t*^BuOO^*t*^Bu^d^	60%	30%
9	air	*m*-CPBA^d^	---^e^	---^e^
10	air	BQ^d^	---^e^	---^e^

a Isolated yields.

b KF (0.4 mmol).

c 79% with KF (0.04 mmol), 79% with KF (0.2 mmol), 74% with KF (0.8 mmol).

d 0.2 mmol of additive.

e Starting material recovered.

f Reaction conditions: cyclopropenone (0.2 mmol), S powder (0.4 mmol), DMF (2.0 mL).

The reaction was found to benefit from KF addition, both under N_2_ (Table 1, entry 3 vs entry 1) and air atmospheres (Table 1, entry 4 vs entry 1) while maintaining the regioselectivity. This observation suggests that elemental sulfur can be activated by a fluoride anion. Further experiments using cyclopropenone **1** with varying KF loadings—catalytic (20 mol %), stoichiometric (1 equiv), and excess (4 equiv) relative to **1**—demonstrated that the reaction proceeds efficiently across all conditions, affording product **3** in 74–79% yields (Table 1, note c). The yields obtained under Wu’s optimized conditions (entry 4) align well with those previously reported for alkylic and aromatic cyclopropenone (72% and 33%, respectively), further validating the robustness of the method.

Interestingly, the addition of TEMPO (1 equiv) increased the reaction yield under both air and N_2_ atmospheres, compared to the corresponding reactions without additives (Table 1, entries 5 vs 1 and 6 vs 2). However, increasing the amount of TEMPO to 5 equiv—the amount typically used as a radical scavenger—did not further improve the yield (72% yield from 2-tridecylcycloprop-2-ene-1-one, data not shown in Table 1).

In contrast, the use of ^*t*^BuOO^*t*^Bu had no noticeable impact on the reaction outcome (Table 1, entries 7 vs 1 and 8 vs 2). This result is consistent with the known thermal stability of di-*tert*-butyl peroxide, which requires temperatures above 100 °C for the homolysis of the O–O bond, rendering it inactive as a radical initiator under the room-temperature conditions employed.

On the other hand, oxidants such as BQ or *m*-CPBA completely suppressed the reaction, leading to full recovery of the starting material (Table 1, entries 9–10). This inhibition is likely due to oxidation of elemental sulfur and/or sulfur impurities in the absence of fluoride salts, preventing its participation in the transformation. However, when KF is present, this inhibitory effect is not observed, presumably due to the facile activation of sulfur by the fluoride ion, thereby overriding the independent activity of these oxidants.

Based on the experimental results obtained, it can be inferred that

a) The reaction efficiently proceeds even in the absence of additives under a nitrogen atmosphere.
b) Molecular oxygen has no significant impact on the reaction outcome; however, oxidants such as BQ or *m*-CPBA completely inhibit the reaction.
c) Fluoride salts enhance the reactivity of sulfur, leading to improved yields.
d) The complete regioselectivity of the reaction is preserved across all tested conditions, consistently yielding the same regioisomer reported by Wu^8^ in the presence of fluoride ions.

### Computational Study of the Reactivity of Elemental Sulfur S_8_

To gain further insight into the underlying reactivity and address the remaining mechanistic uncertainties, a computational study was undertaken. This study was carried out at the ωB97X–D/aug–cc–pV­(T + d)­Z, SMD//ωB97X–D/6–31g­(d,p), and SMD level (see Computational Details), and all energies reported correspond to free energies in solution (1 M, 298.15 K).

The primary substrate investigated was 2-phenylcycloprop-2-en-1-one (**2**), selected for its structural simplicity, inherent asymmetry, and availability of experimental data on its regioselectivity. Various plausible reaction pathways were systematically evaluated, as outlined below. The most stable form of elemental sulfur is singlet cyclooctasulfur, ^**1**^ **S**_**8**_, the corresponding triplet with cyclic structure being highly destabilized (Figures 1 and S10 in the Supporting Information). The minimum corresponding to lineal S_8_, resulting from the opening of the cycle, could not be located on the singlet surface, while the corresponding triplet ^**3**^ **lin-S**_**8**_ is located at 33.4 kcal·mol^–1^ above ^**1**^ **S**_**8.**_ A minimum energy crossing point (^1,3^MECP) at 43.7 kcal·mol^–1^ above ^**1**^ **S**_**8**_ connects this cyclic species with ^**3**^ **lin-S**_**8**_, and corresponds to the opening/closure of the sulfur ring (Figure S10). The high energy required for ring opening supports the idea that elemental sulfur ^**1**^ **S**_**8**_ cannot be cleaved under the reaction conditions without an activating agent.

**Figure 1:**
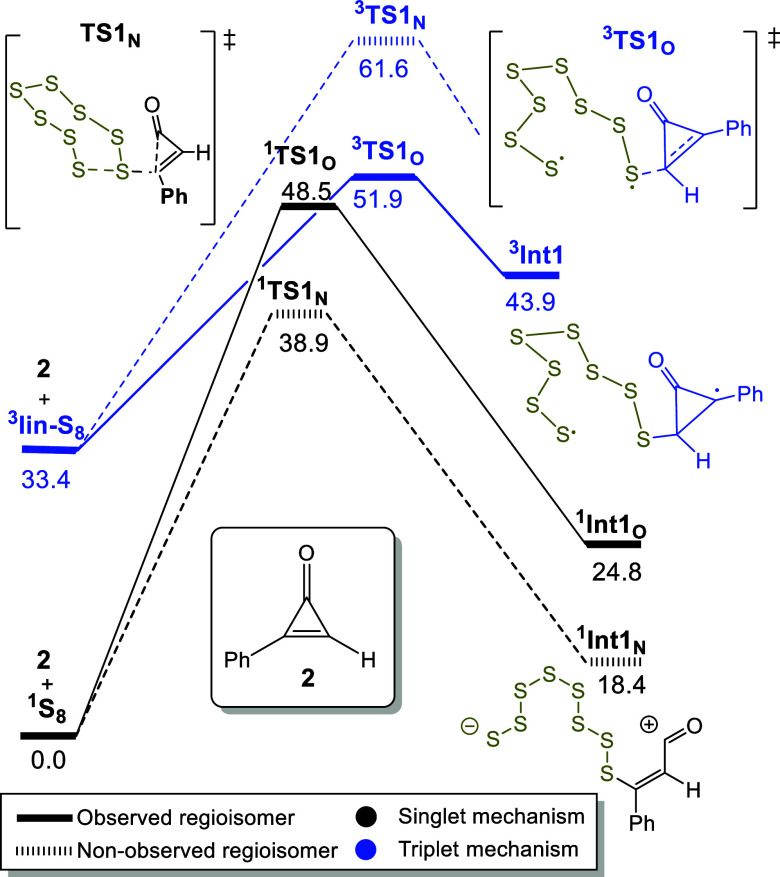
Free-energy profile for the addition of cyclooctasulfur to 2-phenylcycloprop-2-en-1-one (**2**), analogous to Kilmova’s mechanistic proposal. Singlet mechanism is presented in black, while the triplet is presented in blue. Solid lines represent paths leading to the observed regioisomer, while dashed lines represent paths leading to the nonobserved regioisomer. Relative free energies in solution are in kcal·mol^–1^ (25 °C, 1 M).

The computational results reveal the inherent inertness of ^**1**^ **S**_**8**_ (see above) by analyzing the feasibility of different pathways for the reaction between ^**1**^ **S**_**8**_ and cyclopropenone **2**. The calculated free-energy barriers for the conjugated addition of ^**1**^ **S**_**8**_ to substrate **2**, leading to the experimentally observed regioisomer (**4**), and nonobserved alternative are 48.5 and 38.9 kcal·mol^–1^, respectively (Figures 1 and S11 in Supporting Information). Such high barriers render the process unfeasible at room temperature. Direct nucleophilic addition of ^**1**^ **S**_**8**_ to the carbonyl group was also evaluated similarly unfavorable (see Supporting Information, Figure S17), as well as participation of open shell species like triplet ^3^S_8_ or ^3^S_2_ (see Figures 1, S11, and S12 in the Supporting Information). These findings suggest the existence of an unidentified activation pathway that allows elemental sulfur to react under mild conditions. Consequently, the results call into question previously proposed mechanisms (Scheme 2a,b) reliant solely on inactivated ^**1**^ **S**_**8**_ and underscore the need for further investigation to elucidate alternative activation processes.

### Alternative Pathways for Sulfur Activation

Since our studies on inactivated elemental sulfur revealed high activation barriers for its reaction with cyclopropenones, via either 1,2 or 1,4 addition pathways, we performed additional experiments (see Table 2).^12^ Elemental sulfur is known to contain various sulfide impurities that would be capable of initiating sulfur-ring opening,^5b,13^ which could account for the observed reactivity in the absence of added activators (59% for compound **1**, Table 1, entry 1). This hypothesis was partially confirmed experimentally: when highly purified sulfur—either washed with water and freeze-dried or crystallized and sublimed—was used, the yield decreased from 59% (Table 2, entry 1) to 32%–30% (Table 2, entries 2 and 3). The use of crystallized and subsequently sublimed sulfur in combination with catalytic amounts of sodium sulfide (Table 2, entry 4) resulted in a yield of 60%, which is nearly identical to that obtained using commercial, nonpurified sulfur without additives (59%, Table 2, entry 1). Notably, this approach doubled the yield compared to that obtained with crystallized and sublimed sulfur alone (30%, Table 2, entry 3). Likewise, the addition of catalytic amounts of sodium ethylthiolate significantly improved the yield, increasing it from 30% to 67% (Table 2, entry 5 vs entry 3). Finally, the addition of KF to purified sulfur—whether in the quantity used by Wu (2 equiv) or in catalytic amounts—resulted in comparable yields of 80% in both cases (Table 2, entries 6 and 7), matching those obtained with commercial sulfur (Table 2, entry 8).

**Table 2:**
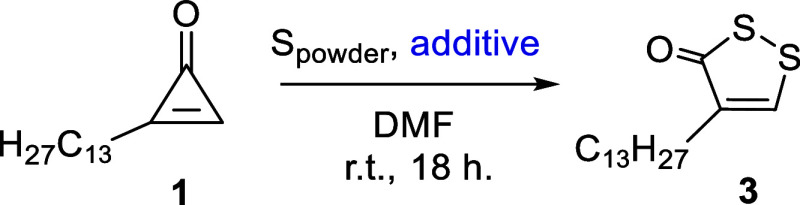
Evaluation of the Impact of Sulfur Impurities on the Reactivity of Cyclopropenone **1** ^b^

entry	S source	Additive	yield^a^ (%)
1	commercial	---	59
2	washed + freeze-dried	---	32
3	crystallized + sublimed	---	30
4	crystallized + sublimed	Na_2_S (20 mol %)	60
5	crystallized + sublimed	CH_3_CH_2_SNa (20 mol %)	67
6	crystallized + sublimed	KF (20 mol %)	80
7	crystallized + sublimed	KF (2 equiv)	80
8	commercial	KF (2 equiv)	77

a Isolated yields.

b Reaction conditions: cyclopropenone (0.2 mmol), S powder (0.4 mmol), DMF (2.0 mL), under N_2_.

These findings demonstrate that sulfur impurities significantly influence the reaction outcome. However, activation by fluoride ions appears to play a predominant role. Nevertheless, since the reaction is not completely suppressed even with highly purified sulfur, it is likely that additional activation pathways beyond the influence of sulfur impurities are operative.

### Reactivity of Polysulfide Anions with Cyclopropenones

Previous computational studies have investigated the nucleophilic ring-opening of cyclic **S**_**8**_ by species such as cyanide or phosphines.^14^ The activation of sulfur by fluoride—leading to highly nucleophilic fluoropolysulfide anions—has also been postulated.^15,16^ Additionally, various sulfur-containing impurities, such as hydrosulfide (HS^–^) or sulfide (S^2–^) anions,^5b,12^ could facilitate the reaction. Hence, we explored the reaction mechanism by considering the singlet fluorooctasulfide anion (^**1**^ **FS**_**8**_^**–**^), and subsequently we extended the study to other fluorosulfide species: fluoroheptasulfide anion (^**1**^ **FS**_**7**_^**–**^), fluorodisulfide anion (^**1**^ **FS**_**2**_^**–**^), and fluorosulfide anion (^**1**^ **FS** ^**–**^), which could plausibly arise from F^–^ and ^**1**^ **S**_**8**_. Additionally, we examined species such as nonasulfanide anion (^**1**^ **HS**_**9**_^**–**^), the trisulfanide anion (^**1**^ **HS**_**3**_^**–**^), and nonasulfanediide anion (^**1**^ **S**_**9**_^**2–**^), which could result from the reaction of hydrosulfide (HS^–^) or sulfide (S^2–^) impurities with ^**1**^ **S**_**8**_. Notably, we demonstrated that both inorganic and organic sulfide salts promote the transformation (see entries 4 and 5 in Table 2). As discussed in detail below, the computed reactivity profiles of all of the analyzed sulfide anions are similar. Thus, while other polysulfide anions might also form under the reaction conditions, their reactivity is likely to be comparable to that of the species studied here.

For the (3 + 2) cycloaddition of cyclopropenone derivatives with fluoropolysulfide anions, we explored the mechanistic proposal previously suggested by Mlostoń. The postulated intermediate **VI**, which results from the nucleophilic attack of the sulfide to the carbonylic carbon (Scheme 2), was found to be very destabilized (Supporting Information, Figure S15). Consequently, we investigated alternative mechanistic pathways. For all polysulfide ions studied, we identified a new mechanism that is fully consistent with the observed regioselectivity, leading to compound **4**. In the main text, the results for ^**1**^ **FS**_**8**_^**–**^ are discussed in detail, while the findings for other polysulfides are summarized, with full computational data provided in the Supporting Information. Considering ^**1**^ **FS**_**8**_^**–**^, the mechanism begins with the conjugate addition of this anion to the less hindered carbon of the cyclopropenone’s C–C double bond, forming **FS**_**8**_ **-** ^**1**^ **Int1**_**O**_, which is 18.2 kcal·mol^–1^ higher in energy than reactants (Figure 2). This intermediate is formed via the transition state **FS**_**8**_–^**1**^ **TS1**_**O**_, located at 23.2 kcal·mol^–1^ above the reactants (Figure 2). Alternatively, addition to the more hindered carbon would lead to a nonobserved product via transition state **FS**_**8**_–^1^ **TS1**_**N**_, located at 26.2 kcal·mol^–1^, less favorable by 3.0 kcal·mol^–1^.

**Figure 2:**
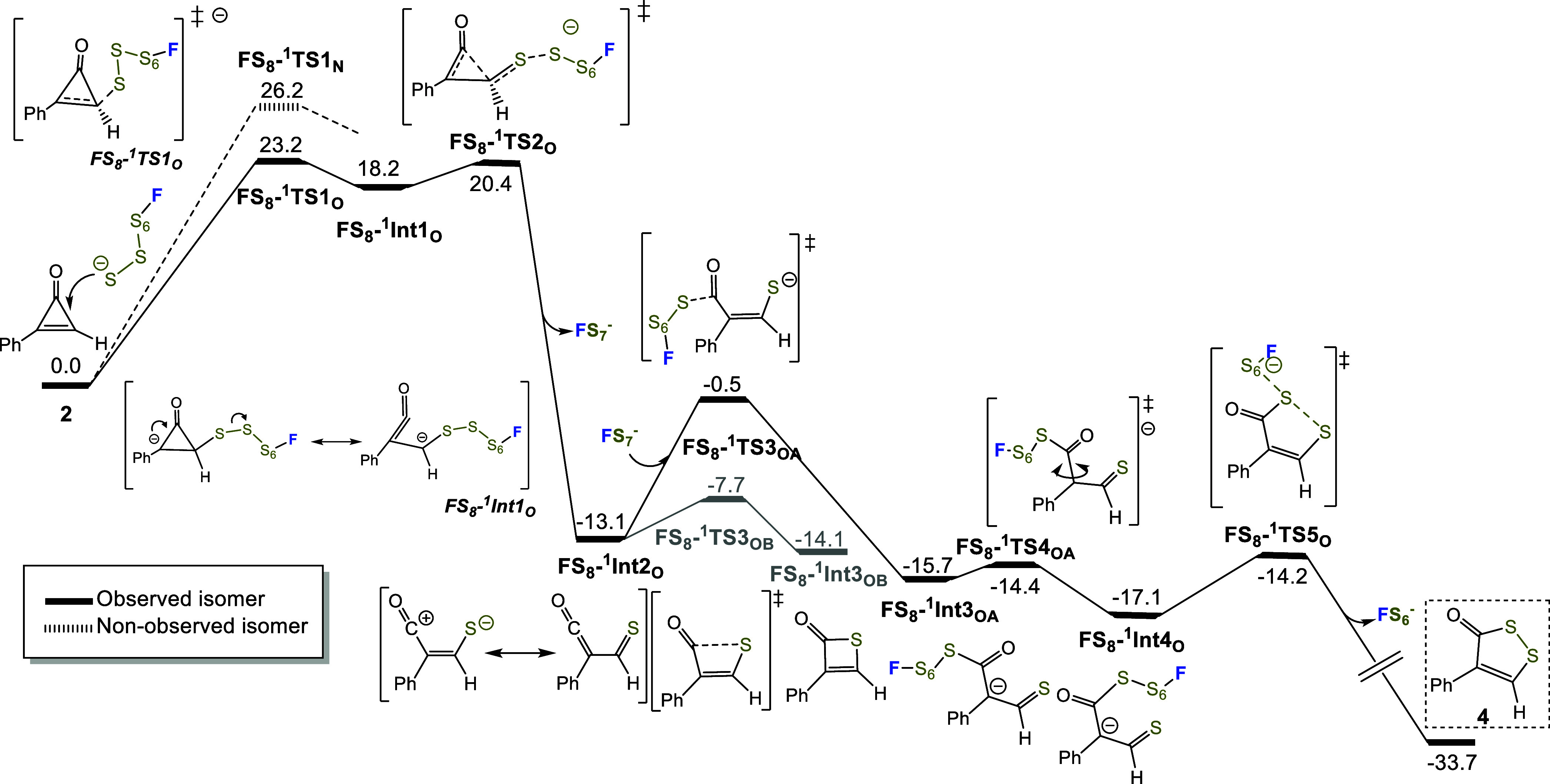
Free energy profile for the formal (3 + 2) cycloaddition of 2-phenylcycloprop-2-en-1-one and fluorooctasulfide anion (**FS**_**8**_^**–**^). Dashed lines correspond to the reaction pathways leading to the experimentally nonobserved regioisomer, and solid ones to the observed regioisomer **4**.^18^ For a complete profile with competing paths, see Figure S24. Energies correspond to relative free energies in solution in kcal·mol^–1^.

**Figure 3:**
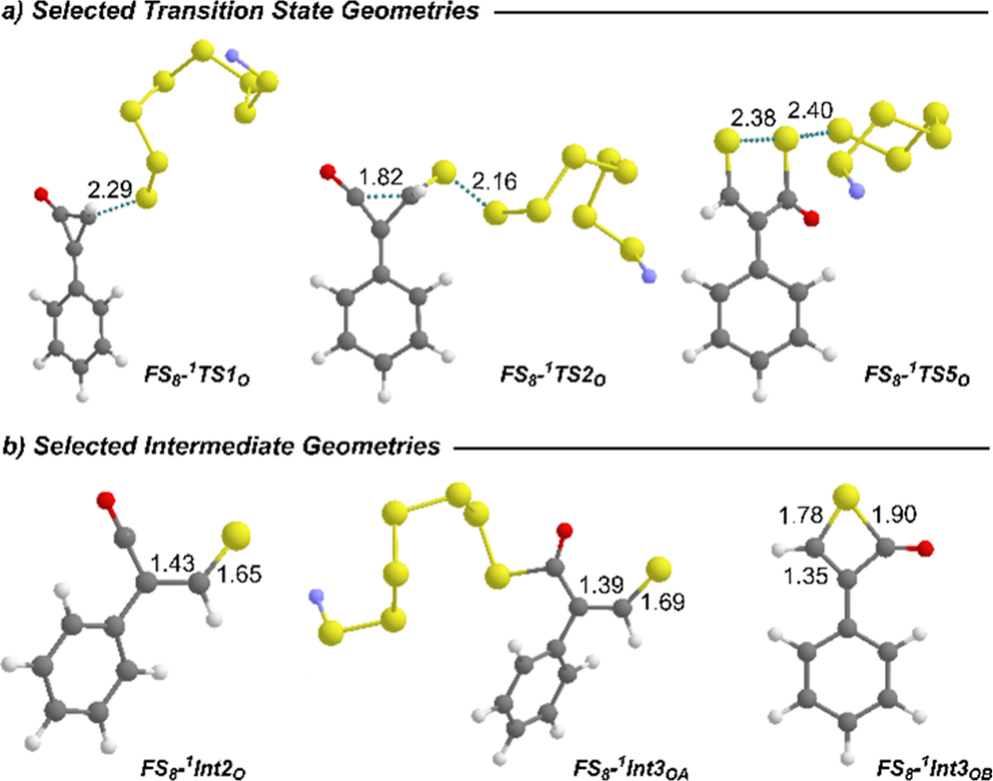
Ball and stick representations of (a) key transition states and (b) key intermediates considering the reaction between ^**1**^ **FS**_**8**_^**–**^ and **2**. Color code: S in yellow, C in gray, O in red, F in violet, and H in white. Distances in Angstrom (Å).

The high energy intermediate **FS**_**8**_ **–** ^**1**^ **Int1**_**O**_ presents C–C distances of 1.34, 1.49, and 1.58 Å and evolves to the low-energy ketenethialdehyde intermediate **FS**_**8**_ **–** ^**1**^ **Int2**_**O**_ via ring opening and extrusion of fluoroheptasulfide ion through **FS**_**8**_ **–** ^**1**^ **TS2**_**O**_ at 20.4 kcal·mol^–1^. Intermediate **FS**_**8**_ **–** ^**1**^ **Int2**_**O**_ is structurally closer to the ketenethialdehyde but with some character of thiolate carbocation (see Figures 2 and 3), with C–C and C–S distances of 1.43 and 1.65 Å, respectively. Hence, both selectivity and rate are governed by the initial step of the reaction. The 23.2 kcal·mol^–1^ barrier is consistent with a reaction occurring at room temperature over approximately 12 h.

Subsequently, **FS**_**8**_ **–** ^**1**^ **Int2**_**O**_ undergoes electrocyclization to afford a thiet-2-one intermediate **FS**_**8**_ **–** ^**1**^ **Int3**_**OB**_ in agreement with that reported by Wentrup.^17^ Both the open **FS**_**8**_ **–** ^**1**^ **Int2**_**O**_ and cyclic thiet-2-one **FS**_**8**_ **–** ^**1**^ **Int3**_**OB**_ intermediates (Figure 3) are highly susceptible to nucleophilic attack to the carbonyl group, ultimately leading to the thioenolate intermediate **FS**_**8**_ **–** ^**1**^ **Int4**_**O**_. Among these pathways, nucleophilic attack to the open **FS**_**8**_ **–** ^**1**^ **Int2**_**O**_ is the most favorable, forming a C–S bond yielding intermediate **FS**_**8**_ **–** ^**1**^ **Int3**_**OA**_, with a barrier of just 12.6 kcal·mol^–1^ (see Figures 2 and 3). This intermediate can then lead to **FS**_**8**_ **–** ^**1**^ **Int4**_**O**_ through a low-barrier C–C bond rotation. Thioenolate **FS**_**8**_ **–** ^**1**^ **Int4**_**O**_ would then afford experimentally obtained regioisomer **4** via nucleophilic ring closure and fluorohexasulfide displacement. Competing alternative pathways are detailed in the Supporting Information (Figure S24).

Attempts to probe the reaction mechanism through detection of the **FS**_**8**_ **–** ^**1**^ **Int2**_**O**_ or **FS**_**8**_ **–** ^**1**^ **Int3**_**OB**_ intermediates by ^1^HNMR, mass spectrometry, or infrared spectroscopy were unsuccessful. As emphasized by Wentrup, the synthesis and observation of highly reactive species such as thiet-2-one or ketenethialdehyde are extremely challenging.^17,19^ Based on the computed activation barriers for the consumption of **FS**_**8**_ **–** ^**1**^ **Int2**_**O**_ and **FS**_**8**_ **–** ^**1**^ **Int3**_**OB**_ and assuming a first or pseudo-first order kinetics, their estimated half-lives range from approximately 1 × 10^–3^ to 2 × 10^–4^ s, rendering their experimental detection highly unlikely.

The KF-mediated mechanism proposed here differs from the previously reported pathways. While it similarly postulates an initial conjugate addition—reminiscent of one of the mechanisms proposed by Klimova^10^ (Scheme 2b)—the subsequent steps diverge entirely, proceeding through the ketenethialdehyde **FS**_**8**_ **–** ^**1**^ **Int2**_**O**_ and the thiet-2-one **FS**_**8**_ **–** ^**1**^ **Int3**_**OB**_ (Figure 3) intermediates. Additionally, this mechanism contrasts with that proposed by Mlostoń^9^ (Scheme 2c), where the fluoropolysulfide anion, generated in the presence of fluoride, reacts through direct addition to the carbonyl group of cyclopropenone.

The computational study of the reaction promoted by fluoride-activated sulfur was extended to other differently substituted cyclopropenones (Table 3). The calculated results are consistent with experimental observations^8^ and provide a rationale for the regioselectivity seen in nonsymmetric cyclopropenones. In all cases, the reaction pathway leading to the experimentally observed regioisomer proceeds through a transition state of lower energy, with the corresponding energy differences relative to the alternative transition state ranging from 2.1 to 6.2 kcal·mol^–1^. When R_1_ is a phenyl group (Table 3, entry 1), the preferred attack occurs at the nonsubstituted carbon, which can be attributed to the greater stabilization of the developing negative charge in the enolate intermediate provided by the aromatic ring. When R_1_ is a phenyl group and R_2_ a methyl group (Table 3, entry 2), the electronic effects of the phenyl group still dictate the regioselectivity. However, the energy difference between the two possible nucleophilic approaches of the sulfur (**^1^TS_1O_** and ^1^ **TS_1N_** is smaller (Table 2, entry 1 vs 2, 3.0 vs 1.5 kcal·mol^–1^) likely due to the increased steric hindrance at the nucleophilic attack site caused by the methyl group relative to hydrogen. For nonsymmetric cyclopropenones substituted with a methyl group (Table 3, entry 3), regioselectivity is influenced by both steric factors and electronic effects. Steric hindrance favors nucleophilic attack at the less substituted carbon of cyclopropenone, while attack at the more hindered double bond carbon provides less stabilization of the developing negative charge in the formation of the enolate. In alkyl-substituted cyclopropenones, steric effects appear to dominate, as indicated by the computational results (Table 3, entry 3). The experimental observation that no significant differences in regioselectivity or yield are observed when the reaction is performed under an inert atmosphere versus in air (Table 1, entry 4 vs entry 3) suggests that the same mechanism proposed above operates in both cases. This underscores the role of fluoride ions in enhancing sulfur reactivity.

**Table 3:**
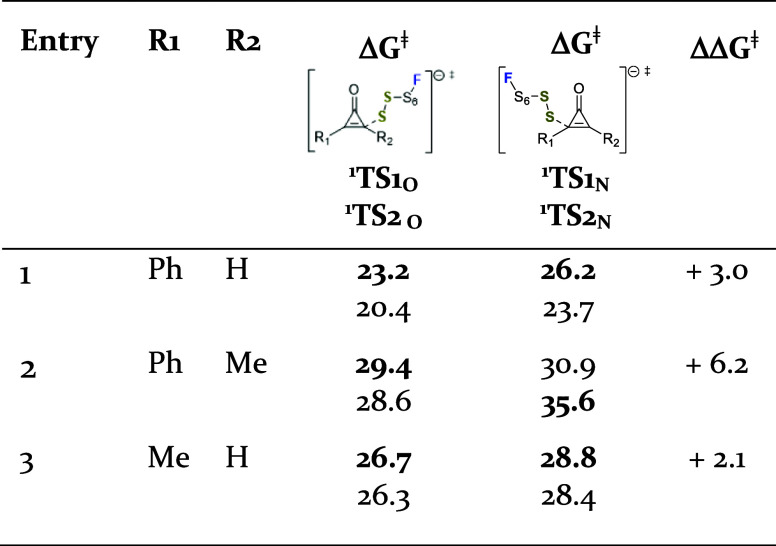
Summary of the Computational Results for the Reaction Promoted by ^**1**^ **FS**_**8**_^**–**^ ^a^ ^b^

a Free-energies in solution in kcal·mol^–1^.

b Δ*G* ^⧧^ is the free energy barrier for the formation of the represented regioisomer, ΔΔ*G* ^⧧^ difference of free energy barriers between the regioisomers.

As mentioned above, the (3 + 2) cycloaddition of 2-phenylcycloprop-2-en-1-one with the fluoroheptasulfide anion (^**1**^ **FS**_**7**_^**–**^) and fluorodisulfide anion (^**1**^ **FS**_**2**_^**–**^) was also examined (see Supporting Information, Figures S17 and S18). These results are consistent with the experimentally observed regioselectivity. A mechanism similar to that proposed for the fluorooctasulfide anion (^**1**^ **FS**_**8**_^**–**^) was identified, but with somewhat lower overall barriers of 21.5 and 17.6 kcal·mol^–1^, respectively. These findings indicate that different fluoropolysulfide anions formed upon fluoride activation of S_8_ can also react with cyclopropenones and that the shorter the sulfur chain, the lower the energy barrier. In general, species generated either by fluoride activation or during the course of the reaction can attack the starting cyclopropenone or the thiet-2-one intermediate. The same mechanism was also found for other polysulfides studied. Nonasulfanide (^**1**^ **HS**_**9**_^**–**^), trisulfanide (^**1**^ **HS**_**3**_^**–**^), and nonasulfanediide (^**1**^ **S**_**9**_^**2–**^) anions showed overall barriers of 17.3, 15.8, and 18.6 kcal·mol^–1^, respectively (see Supporting Information, Figures S21–S23), all somewhat lower than that of ^**1**^ **FS**_**8**_^**–**^. These results indicate that all tested polysulfide anions can undergo the cycloaddition. All in all, the proposed mechanism shown in Figure 2 explains the efficiency of the reaction with catalytic amounts of KF or impurities as the expelled polysulfide species (i.e., FS_*n*‑2_^–^, HS_*n*‑2_^–^, and S_*n*‑2_^2–^species) can react again with a new cyclopropenone or activate new elemental sulfur molecules.

Finally, it is interesting to note that the mechanism involving triplet disulfur (^**3**^ **S**_**2**_), presented in the Supporting Information (Figure S12), also accounts for the observed regioselectivity. If accessible, the reaction could proceed on the triplet surface. However, our calculations indicate that ^**3**^ **S**_**2**_ is not readily generated or available under the experimental conditions. The efficient progression of the reaction in the presence of the radical scavenger TEMPO is consistent with these computational results, allowing us to rule out radical-type pathways.

## Conclusions

The reaction of nonsymmetrically substituted cyclopropenones with elemental sulfur proceeds without additives under both nitrogen and air, delivering complete regioselectivity. The addition of KF improves efficiency by enhancing yields, particularly under substoichiometric conditions, while preserving the regioselectivity. Oxidants such as *m*-CPBA and BQ inhibit the reaction in the absence of KF. However, when KF is present, sulfur activation proceeds efficiently, masking the individual effects of these additives.

The computational study indicates that mechanisms relying solely on cyclic S_8_ molecules of elemental sulfur are unlikely under the mild conditions employed. Therefore, the true reactive species are polysulfides that are produced by fluoride salts, sulfide salts, or sulfide impurities of elemental sulfur, which can initiate an S_8_ ring opening.

The results obtained using various purified forms of elemental sulfur suggest that this type of control experiment may be advisable when reporting reactions involving the use of S_8_.

The proposed mechanism involves the presence of soft nucleophilic polysulfide anions (^**1**^ **FS**_***n***_^**–**^, ^**1**^ **S**_***n***_^**2–**^, and ^**1**^ **HS**_***n***_^**–**^); and has two main steps: (1) nucleophilic attack of polysulfide anions to the cyclopropenone C=C π* bond at the less substituted carbon; and (2) sequential transfer of the two sulfur atoms through the formation of 1,2-dithiol-3-one intermediate. The reaction’s efficiency in the presence of TEMPO, together with computational results, rules out radical pathways.

These findings challenge previously proposed mechanistic models and underscore the crucial role of polysulfide ions generated with fluoride or sulfide salts or from trace impurities, providing new insights into the reactive forms of elemental sulfur as a reagent in organic transformations. Beyond these fundamental mechanistic contributions, the anion-mediated activation of elemental sulfur S_8_ can also have practical relevance in applied contexts, such as sulfur vulcanization processes.^20^

## Experimental Section

General methods, synthetic procedures, and characterization data are included in the Supporting Information.

## Supplementary Material



## Data Availability

The data underlying this study are available in the published article and its Supporting Information.

## Notes

Methods and synthetic procedures, characterization data, NMR spectra, computational study, complementary data and optimized geometries, and energies of relevant species (PDF)
