# Supplementary material for: Mechanistic Insights into the Regioselective (3 + 2) Cycloaddition of Unsymmetrical Cyclopropenones with Elemental Sulfur: Experimental and Computational Studies
Source: J Org Chem. 2026 Jan 14;91(4):1485–92. doi: 10.1021/acs.joc.5c01955 (PMC12865767; doi:10.1021/acs.joc.5c01955)
Supplement: Supplementary file 1 [file jo5c01955_si_001.pdf]

SUPPORTING INFORMATION

Mechanistic Insights into the Regioselective (3+2) Cycloaddition of Unsymmetrical Cyclopropenones with Elemental Sulfur: Experimental and Computational Studies

Pablo Rivero,[a‡] Gonzalo D. Nuñez,[b‡] Eric Miró,[a‡] Mario Villares,[b‡] Jorge J. Carbó,*[b] Sergio Castillón,[a] Yolanda Díaz,*[a] Maria Besora,*[b] María Isabel Matheu.*[a]

[a] Dr. P. Rivero, E. Miró, Prof. S. Castillón, Dr. Y. Díaz, Prof. M.I. Matheu
Departament de Química Analítica i Química Orgànica, Faculty of Chemistry
Universitat Rovira i Virgili
C/ Marcel·lí Domingo 1, 43007, Tarragona (Spain).
E-mail: maribel.matheu@urv.cat; yolanda.diaz@urv.cat

[b] G. D. Nuñez, M. Villares, Dr. M. Besora, Prof. J. J. Carbó.
Departament de Química Física i Inorgànica, Faculty of Chemistry
Universitat Rovira i Virgili
C/Marcel·lí Domingo 1, 43007, Tarragona (Spain).
E-mail: j.carbo@urv.cat; maria.besora@urv.cat

‡ These authors contributed equally

Contents

1. General information S3
   1.1. General synthetic methods S3
   1.2. Safety Considerations S3
   1.3. Computational Details S3
2. Synthetic procedures and product characterization S5
3. NMR Spectra S7
4. Computational Study Complementary Data S12
   4.1. Self-activation of S_8 .... S12
   4.2. Reaction mechanism for the (3+2) cycloaddition of 2-phenylcycloprop-2-en-1-one, **2**, with inactivated cyclic $^1S_8$. S13
   4.3. Reaction mechanism for the (3+2) cycloaddition of 2-phenylcycloprop-2-en-1-one, **2**, with triplet disulfur $^3S_2$. S14

4.4. Previous postulated mechanisms S15

4.5. Reaction mechanism for the (3+2) cycloaddition of 2-phenylcycloprop-2-en-1-one, **2**, with fluoroheptasulfide anion, FS_7^-. S18

4.6. Reaction mechanism for the (3+2) cycloaddition of 2-phenylcycloprop-2-en-1-one, **2**, with fluorodisulfide anion, FS_2^-. S19

4.7. Reaction mechanism for the (3+2) cycloaddition of 2-phenylcycloprop-2-en-1-one, **2**, with fluorosulfide anion, FS^-. S21

4.8. Reaction mechanism for the (3+2) cycloaddition of 2-phenylcycloprop-2-en-1-one, **2**, with nonasulfanide anion, HS_9^-. S22

4.9. Reaction mechanism for the (3+2) cycloaddition of 2-phenylcycloprop-2-en-1-one, **2**, with trisulfanide anion, HS_3^-. S23

4.10. Reaction mechanism for the (3+2) cycloaddition of 2-phenylcycloprop-2-en-1-one, **2**, with nonasulfanediide anion, S_9^-. S24

4.11. More details on the reaction mechanism for the [3+2] cycloaddition of 2-phenylcycloprop-2-en-1-one, 2, with fluorooctasulfide anion, FS_8^-. S25

5. Optimized Geometries and Energies of Relevant Species S27

6. References S36

1. General information

1.1. General synthetic methods

All reactions sensitive to air and/or moisture were carried out in anhydrous conditions: performing vacuum-argon cycles in the flasks to be used, previously dried heating with a heating gun under vacuum, as well as transferring the reagents and solvents with cannulas or syringes previously purged with argon. The procedures described below are the ones that performed with the highest yield.

High–resolution mass spectra (HRMS) were recorded on an Agilent 1100 Series LC/MSD mass spectrometer with electrospray ionization (ESI). Exact m/z values are reported in Daltons. ^{1}H and ^{13}C NMR spectra were obtained with a Varian Mercury VX 400 and visualized with the MestreNova software (MestreLab®).

The coupling constants (*J*) are described in Hz using the following abbreviations: s = singlet, d = doublet, t = triplet, q = quadruplet, m = multiplet, ap = apparent, br = broad. All spectra are referenced in relation to the residual signal of the deuterated solvent used.

Thin-layer chromatography was performed on 0.25 mm E. Merck® aluminum plates coated with 60 F_{254} silica, using *p*-anisaldehyde or $KMnO_4$ as developer as indicated. Chromatographic columns were performed by passing the mentioned solvent under pressure through Fluka® or Merck® silica gel 60 (230-400 mesh).

1.2. Safety Considerations

All experimental procedures were conducted using standard laboratory practices and reagents that do not present unusual hazards. The work did not involve any unexpected, new, or significant risks beyond those typically associated with routine chemical laboratory operations.

1.3. Computational Details

All calculations were carried out using Gaussian 16[1] suit of programs within the Density Functional Theory (DFT). All structures of minima and transition states were optimized with the ωB97XD functional[2] together with the 6-31g(d,p)[3] basis set for all atoms. The solvent effects were included during optimizations employing the SMD[4] implicit solvation model with Gaussian's default parameters for *N*,*N*-dimethylformamide. After optimizations, single point (SP) calculations were run with the same DFT functional but with the larger aug-cc-pV(T+d)Z[5] basis set for all atoms to improve the description of the energies. All energies presented correspond to Gibbs free-energies resulting from the single points in solution at 298.15 K in kcal·mol^{-1} unless otherwise stated. Free energies were computed as $\Delta G_{SP} = \Delta G_{OPT} - \Delta E_{OPT} + \Delta E_{SP}$, where SP indicates the single points at ωB97XD/aug-cc-pV(T+d)Z+SMD level and OPT the optimization energies at ωB97XD/6-31g(d,p)+SMD level. Standard state corrections (from 1 atm. to 1 M), and quasi-harmonic approximation corrections as proposed by Grimme for the contribution to the entropy[6] of low-lying vibrational modes were applied using the program GoodVives.[7] aug-cc-pV(T+d)Z basis sets were obtained from the Basis Set Exchange (BSE).[8]

Minimum Energy Crossing Points were obtained using Prof. J. N. Harvey program[9] through easyMECP[10]. The free energies at the MECP were obtained by averaging the projected frequencies at the singlet and triplet surfaces.

A data set collection of computational results is available in the ioChem-BD repository.[11]
Access the collection following the link: DOI: 10.19061/iochem-bd-2-86

2. Synthetic procedures and product characterization

2-Tridecylcycloprop-2-en-1-one (1)[12]

NaI (2.526 g, 16.85 mmol) and 1-pentadecyne (2 mL, 7.62 mmol) were dissolved in dry THF (10.2 mL) under argon atmosphere in a Schlenck pressure tube. $TMSCF_3$ (5.63 mL, 38.09 mmol) was added to the solution. The Schlenck pressure tube was sealed and the mixture was heated at 110 ˚C while vigorously stirring. After 21 h $NaHCO_3$ saturated aqueous solution was added to the reaction mixture. Both phases were separated, and the aqueous phase was extracted with Et_2O. The combined organic extracts were desiccated with anhydrous $MgSO_4$ and concentrated under vacuum to give a yellowish oily residue. The obtained residue was dissolved in a hexane/AcOH mixture (99:1, 140 mL) and treated with silica at room temperature overnight. Afterwards, the mixture was filtrated under vacuum through a filter plate and extracted with $NaHCO_3$ saturated solution. The organic phase was desiccated with anhydrous $MgSO_4$ and concentrated under vacuum to give a yellowish oily residue. The residue was purified by flash column chromatography on silica gel (gradient: hexane/ethyl acetate, from 7:3 to 6:4) and finally the desired 2-tridecylcycloprop-2-en-1-one was obtained as a white solid (1.724 g, 7.29 mmol, 96 % yield).

$\boldsymbol{R_f}$ = 0.20 (hexane/ethyl acetate, 1:1); **M.p.** 41-42 °C; **^{1}H NMR** (400 MHz, $CDCl_3$) δ in ppm: 8.40 (s, 1H), 2.64 (t, *J* = 7.3 Hz, 2H), 1.68 (q ap., *J* = 7.4 Hz, 2H), 1.40-1.33 (m, 2H), 1.30-1.17 (m, 20H), 0.84 (t, *J* = 6.6 Hz, 3H); **^{13}C {^{1}H} NMR** (100.6 MHz, $CDCl_3$) δ in ppm: 170.3, 158.0, 148.2, 32.0, 29.70, 29.68, 29.6, 29.5, 29.4, 29.2, 29.0, 27.5, 25.7, 22.7, 14.2; **HRMS** (TOF ES+) for $[M+H]^+$ $C_{16}H_{29}O^+$ (m/z): calculated: 237.2213 found: 237.2212.

2-Phenylcycloprop-2-en-1-one (2)[13]

NaI (2.512 g, 16.8 mmol) and phenylacetylene (0.83 mL, 7.5 mmol) were dissolved in dry THF (10.2 mL) under argon atmosphere in a Schlenck pressure tube. $TMSCF_3$ (5.6 mL, 37.9 mmol) was added to the solution. The Schlenck pressure tube was sealed and the mixture was heated at 110 ˚C while vigorously stirring. After 21 h $NaHCO_3$ saturated aqueous solution was added to the reaction mixture. Both phases were separated, and the aqueous phase was extracted with AcOEt. The combined organic extracts were desiccated with anhydrous $MgSO_4$ and concentrated under vacuum to give a brown oily residue. The crude product was purified by flash column chromatography on silica gel (hexane/ethyl acetate, from 7:3 to 6:4) to obtain 2-phenylcycloprop-2-en-1-one as a light-yellow oil (588.7 mg, 4.5 mmol, 60 % yield).

$\boldsymbol{R_f}$ = 0.20 (hexane/ethyl acetate, 1:1); **^{1}H NMR** (400 MHz, $CDCl_3$) δ in ppm: 8.51 (s, 1H), 7.88-7.85 (m, 2H), 7.64-7.59 (m, 1H), 7.58-7.53 (m, 2H); **^{13}C {^{1}H} NMR** (100.6 MHz, $CDCl_3$) δ in ppm: 162.2, 155.5, 140.9, 133.7, 131.3, 129.5, 123.3; **HRMS** (TOF ES+) for $[M+H]^+$ $C_9H_7O^+$ (m/z): calculated: 131.0491 found: 131.0491.

4-Tridecyl-3*H*-1,2-dithiol-3-one (3)

Cyclopropenone **1** (0.2 mmol) was dissolved in DMF. Powdered sulfur (2.0 equiv.), KF (2.0 equiv), and DMF (2 mL) were added to the solution. The reaction mixture was stirred at room temperature under air atmosphere for 12 h. After this time, reaction mixture was diluted with Et_2O and washed with brine four times. The organic fraction was desiccated with Na_2SO_4, filtrated and concentrated under vacuum to furnish a brown oily residue. The crude product was purified by flash column chromatography on silica gel (hexane/ethyl acetate, 95:5) to provide the corresponding product as a white solid in a 68% yield. The compound was crystallized by slow diffusion of hexane into a solution of the product in ethyl acetate.

***R*$_f$** = 0.49 (hexane/ethyl acetate, 9:1); **M.p.** 58.5-59.0 °C; **^{1}H NMR** (400 MHz, $CDCl_3$) δ in ppm: 7.92 (s, 1H), 2.43 (t, 2H), 1.58-1.55 (m, 2H), 1.32-1.25 (m, 20H), 0.88 (t, 3H); **^{13}C {^{1}H} NMR** (100.6 MHz, $CDCl_3$) δ in ppm: 195.4, 147.1, 136.0, 32.1, 29.8, 29.8, 29.8, 29.7, 29.5, 29.5, 29.4, 28.6, 28.5, 22.8, 14.3; **HRMS** (TOF ES+) for $[M+H]^+$ $C_{16}H_{29}OS_2^+$ (m/z): calculated: 301.1654 found: 301.1656**.**

4-Phenyl-3*H*-1,2-dithiol-3-one (4)[13]

Cyclopropenone **3** (0.2 mmol) was dissolved in DMF. Powdered sulfur (2.0 equiv), KF (2.0 equiv.), and DMF (2 mL) were added to the solution. The reaction mixture was stirred at room temperature under air atmosphere for 12 h. After this time reaction mixture was diluted with Et_2O and washed with brine four times. The organic fraction was desiccated with Na_2SO_4, filtrated and concentrated under vacuum to furnish a brown oily residue. The crude product was purified by flash column chromatography on silica gel (hexane/ethyl acetate, 95:5) to provide the corresponding product **4** as a white solid in a 39% yield.

***R*$_f$** = 0.31 (hexane/ethyl acetate, 1:1); **M.p.** 82.1-82.6 °C; **^{1}H NMR** (400 MHz, $CDCl_3$) δ in ppm: 8.37 (s, 1H), 7.59-7.56 (m, 2H), 7.45-7.36 (m, 3H); **^{13}C {^{1}H} NMR** (100.6 MHz, $CDCl_3$) δ in ppm: 193.4, 150.0, 134.3, 132.0, 129.0, 128.0; **HRMS** (TOF ES+) for $[M+H]^+$ $C_9H_7OS_2^+$ (m/z): calculated: 194.9933 found: 194.9935.

3. NMR Spectra

2-Tridecylcycloprop-2-en-1-one (1)

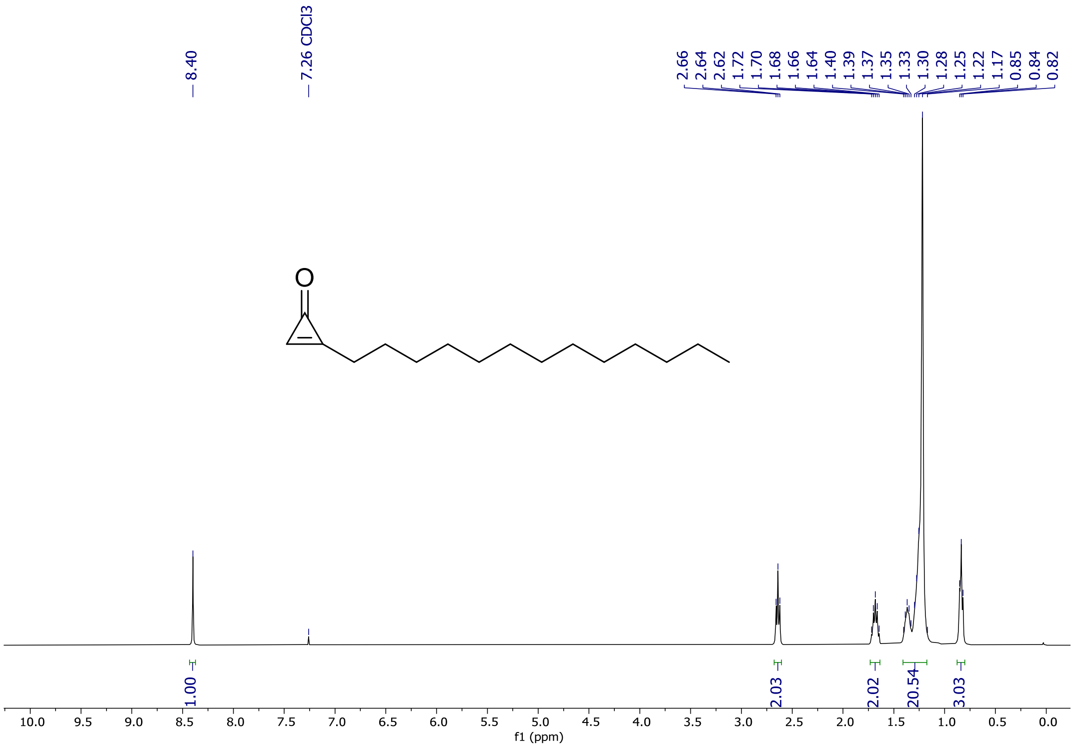

Figure S1. ^{1}H NMR (400 MHz, $CDCl_3$) of 2-tridecylcycloprop-2-en-1-one (**1**)

.

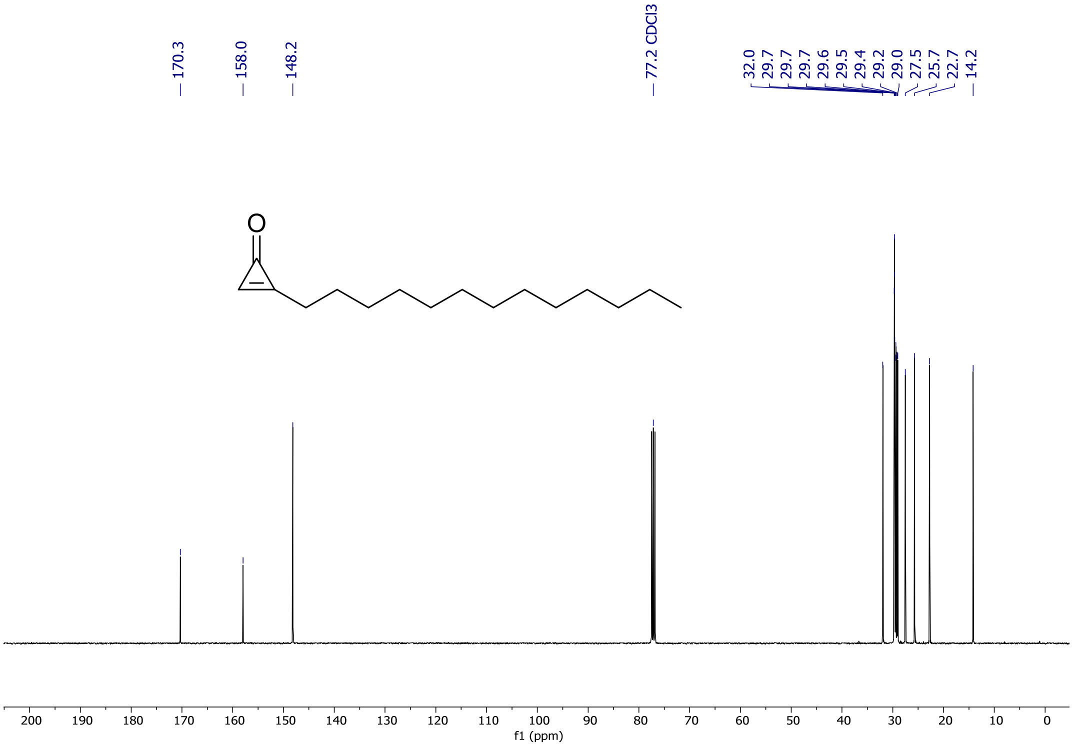

Figure S2. ^{13}C {^{1}H} NMR (100.6 MHz, $CDCl_3$) of 2-tridecylcycloprop-2-en-1-one (**1**)

2-Phenylcycloprop-2-en-1-one (2)

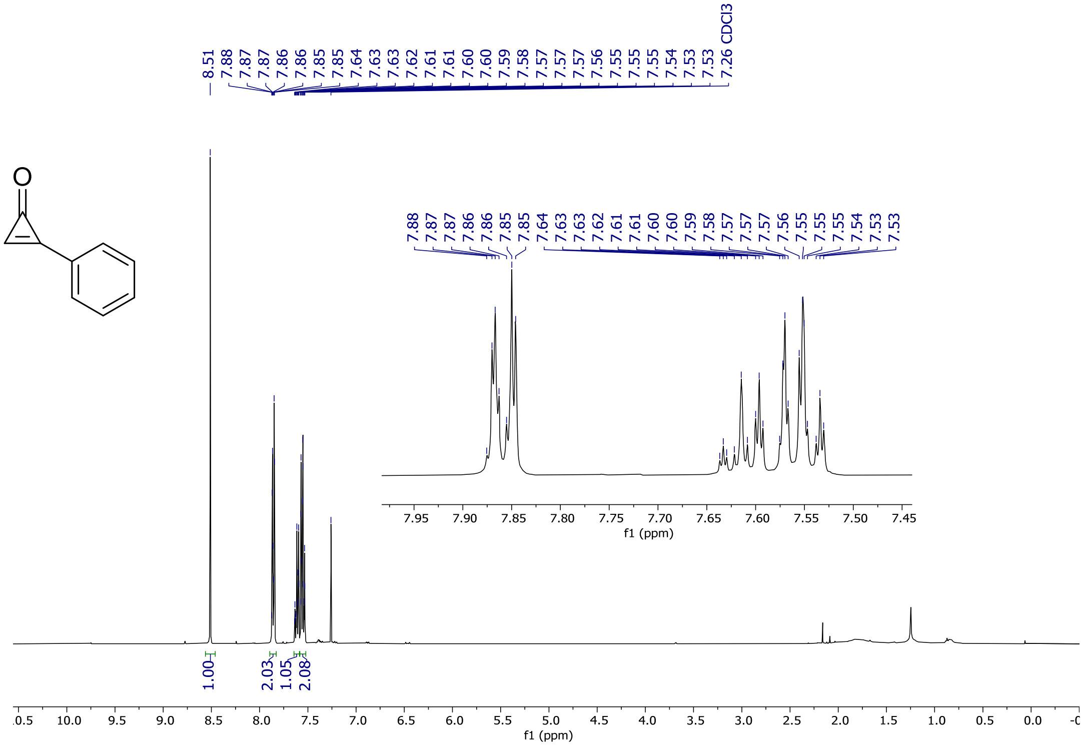

Figure S3. ^{1}H NMR (400 MHz, $CDCl_3$) of 2-phenylcycloprop-2-en-1-one (**2**).

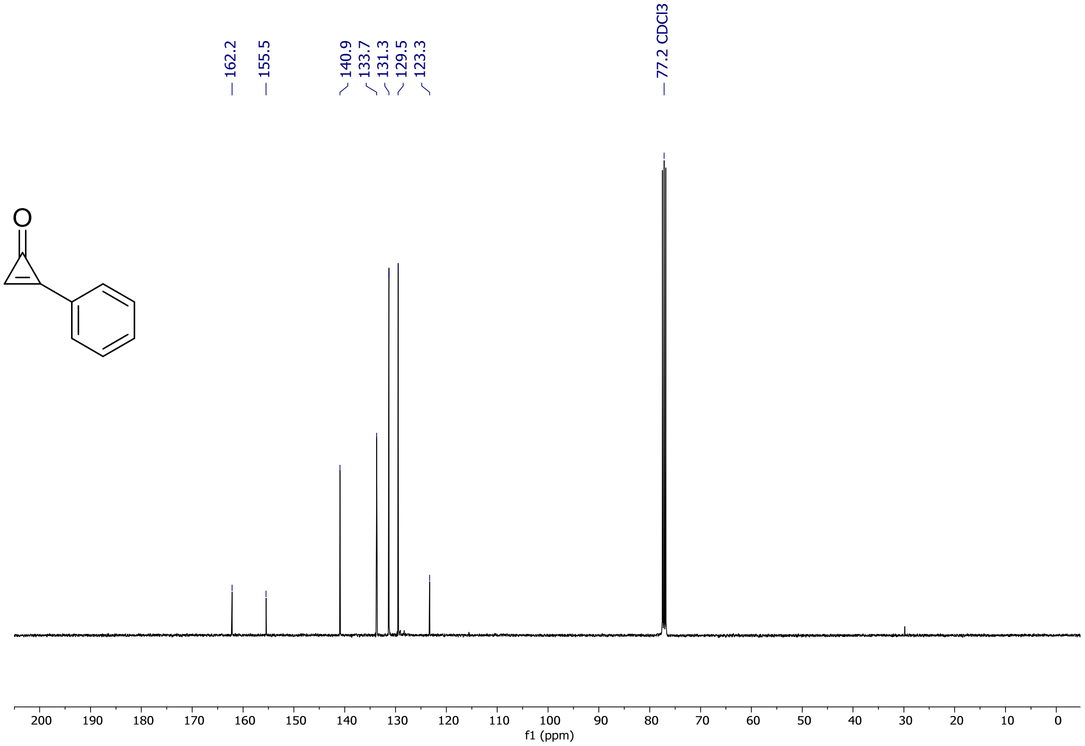

Figure S4. ^{13}C {^{1}H} NMR (100.6 MHz, $CDCl_3$) of 2-phenyllcycloprop-2-en-1-one (**2**).

4-Tridecyl-3*H*-1,2-dithiol-3-one (3)

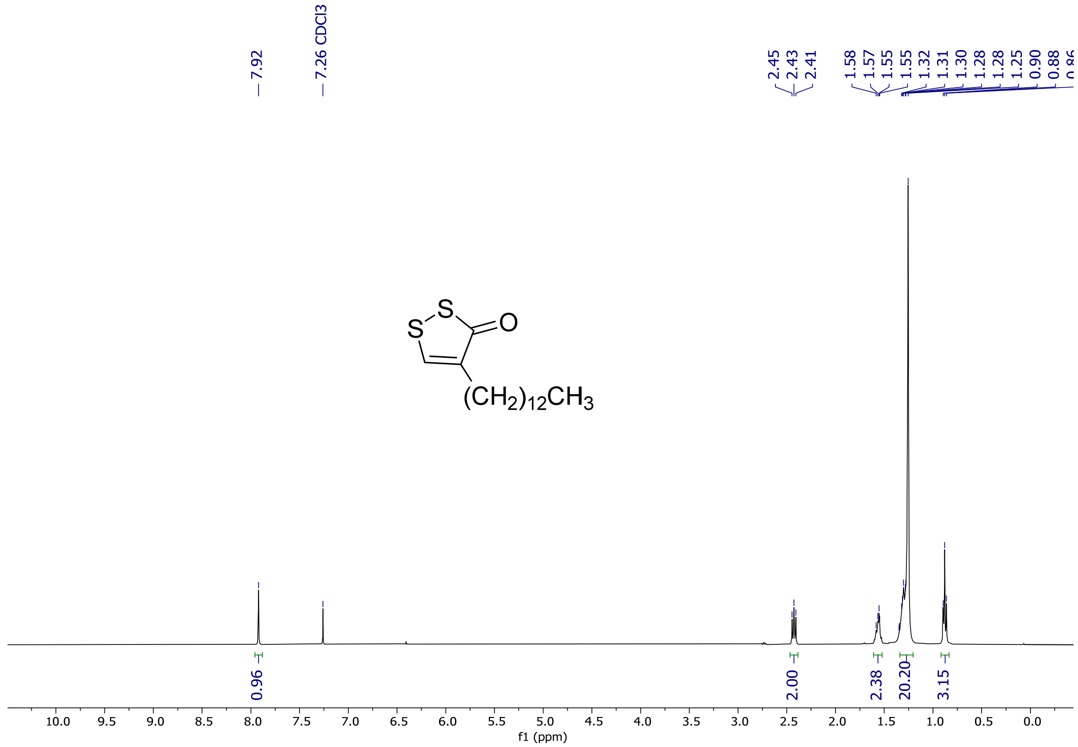

Figure S5. ^{1}H NMR (400 MHz, $CDCl_3$) of 4-tridecyl-3*H*-1,2-dithiol-3-one (**3**).

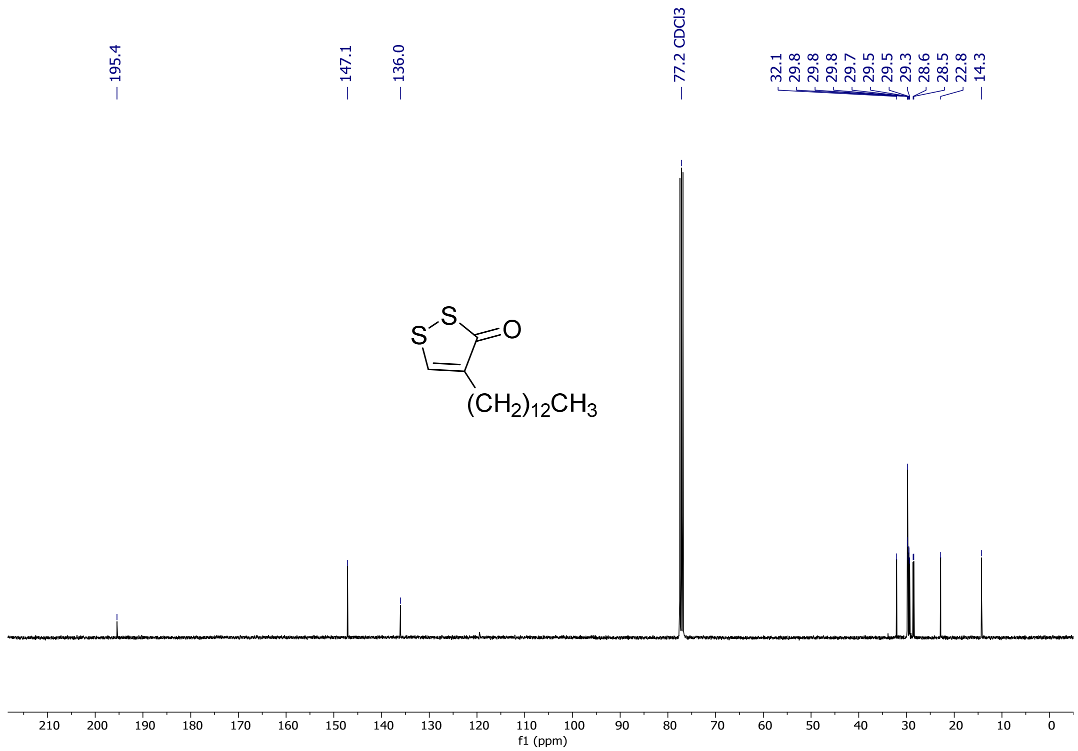

Figure S6. ^{13}C {^{1}H} NMR (100.6 MHz, $CDCl_3$) of 4-tridecyl-3*H*-1,2-dithiol-3-one (**3**).

The unambiguous structural assignment of regioisomer **3** was confirmed by HMBQC analysis, which revealed a clear correlation between the methylene protons attached to the heterocyclic ring and the benzylic carbon (Figure S.7)

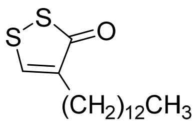

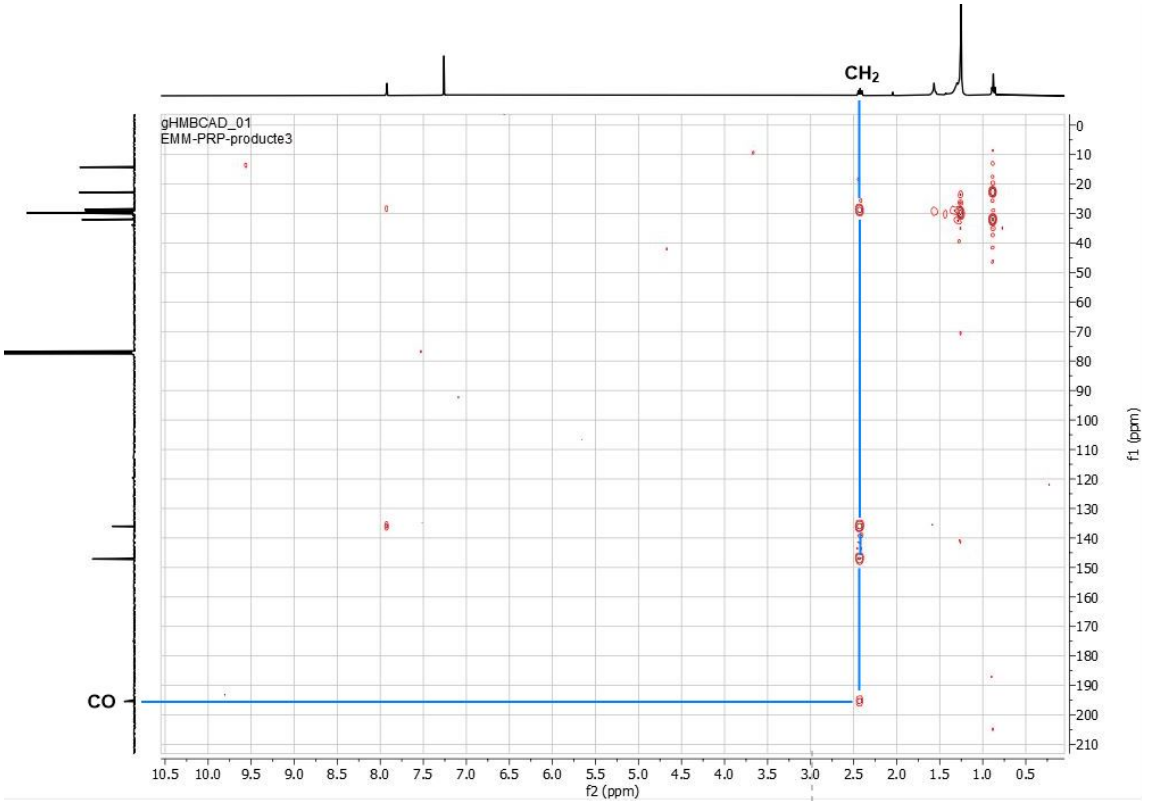

Figure S7. HMBQC of 4-tridecyl-3*H*-1,2-dithiol-3-one (**3**)

4-Phenyl-3*H*-1,2-dithiol-3-one (4)

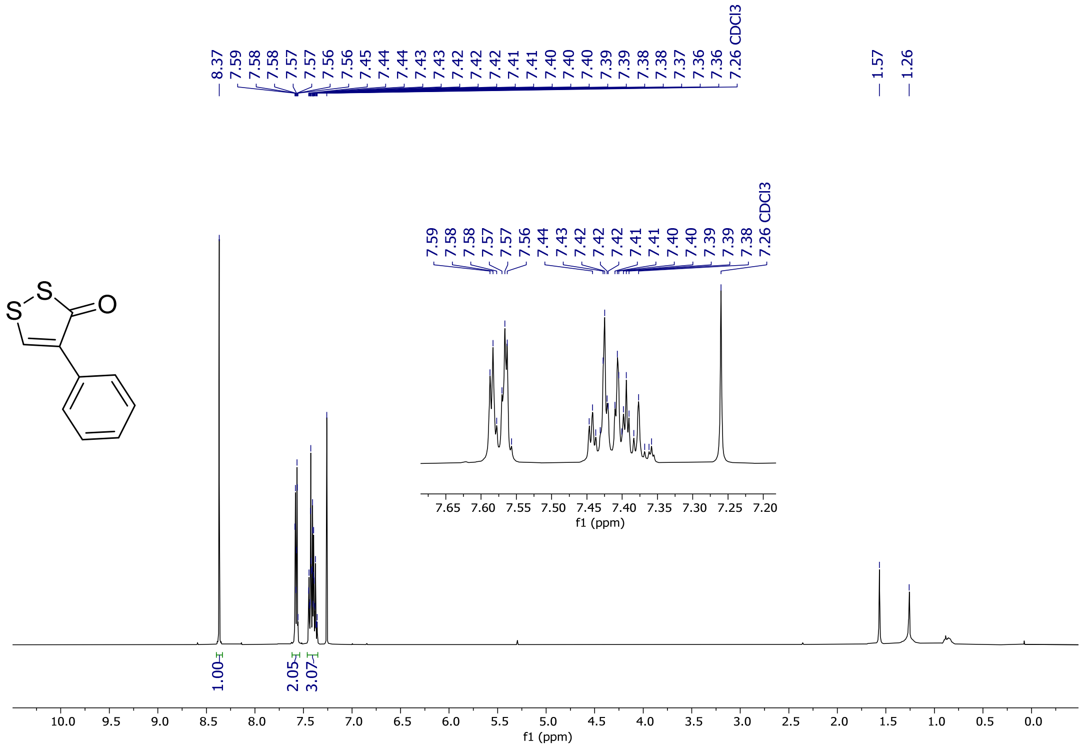

Figure S8. ^{1}H NMR (400 MHz, $CDCl_3$) of 4-phenyl-3*H*-1,2-dithiol-3-one (**4**).

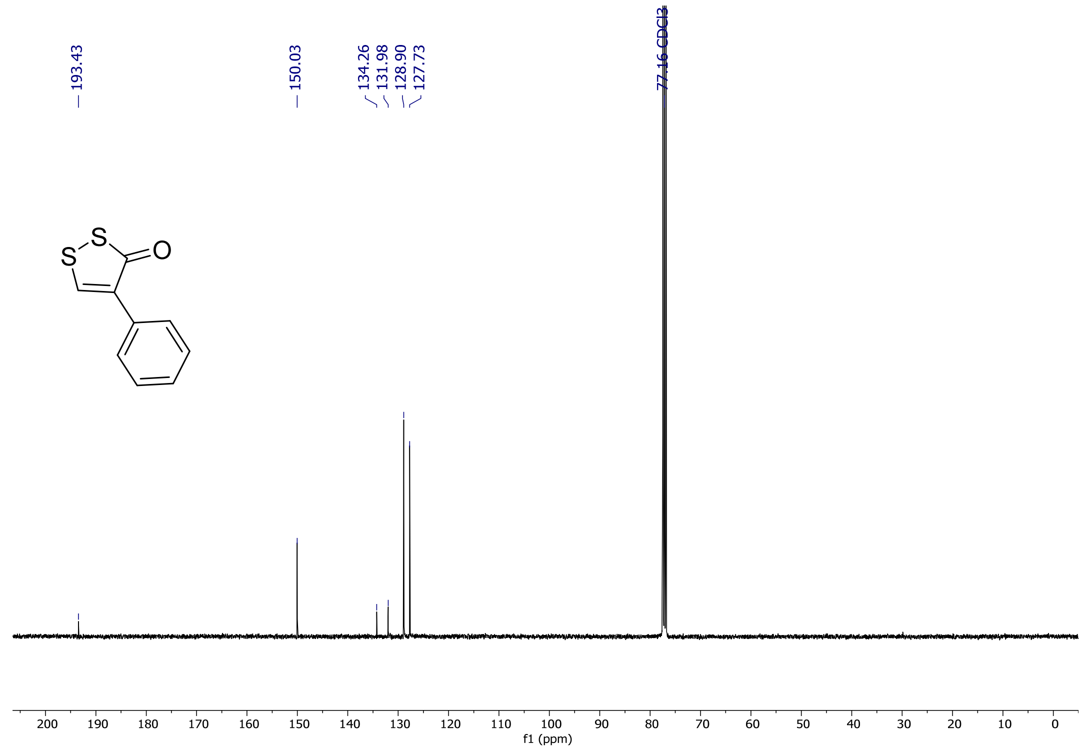

Figure S9. ^{13}C {^{1}H} NMR (100.6 MHz, $CDCl_3$) of 4-phenyl-3*H*-1,2-dithiol-3-one (**4**).

4. Computational Study Complementary Data

4.1. Self-activation of S_8

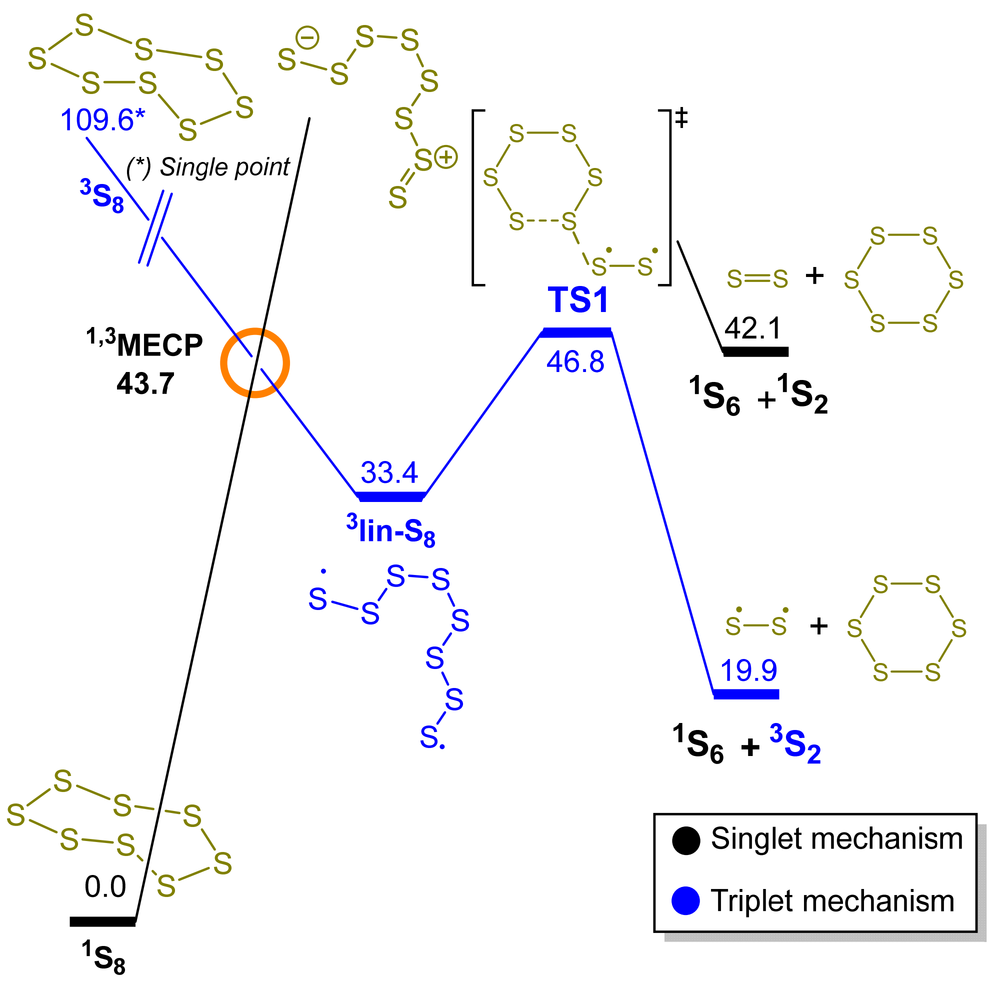

Figure S10. Reactivity of cyclooctasulfur, ^{1}**S$_8$**. Relative free energies in solution and in kcal·mol^{-1} (25 ºC, 1M). * Triplet ^{3}**S$_8$** couldn't be optimized as it opens to form the ^{3}lin-S_8, its energy has been estimated by performing a triplet single point calculation at the geometry of ^{1}**S$_8$**.

4.2. Reaction mechanism for the (3+2) cycloaddition of 2-phenylcycloprop-2-en-1-one, 2, with inactivated cyclic $^1S_8$.

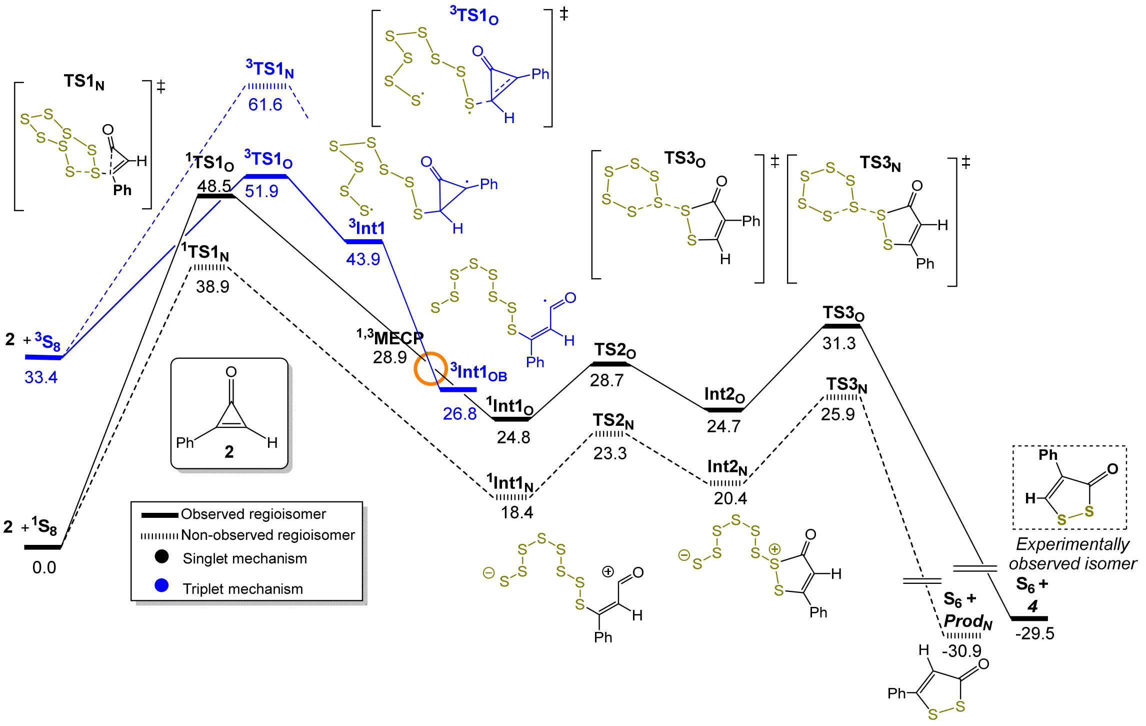

Figure S11. Free energy profile for the reactivity of the 2-phenylcycloprop-2-en-1-one **2** and cyclicoctasulfur, $^1\mathbf{S_8}$. Dashed lines correspond to the reaction pathways leading to the experimentally non-observed regioisomer (***Prod$_N$***), solid ones to the observed regioisomer (***4***). Energies correspond to relative free energies in solution in kcal·mol^{-1} (25 ºC, 1 M).

4.3. Reaction mechanism for the (3+2) cycloaddition of 2-phenylcycloprop-2-en-1-one, 2, with triplet disulfur $^3S_2$.

Please note that in this profile the zero of energy is set on triplet $^3S_2$, a species 19.9 kcal·mol^{-1} less stable than $^1\mathbf{S_8}$ (see Figure S10).

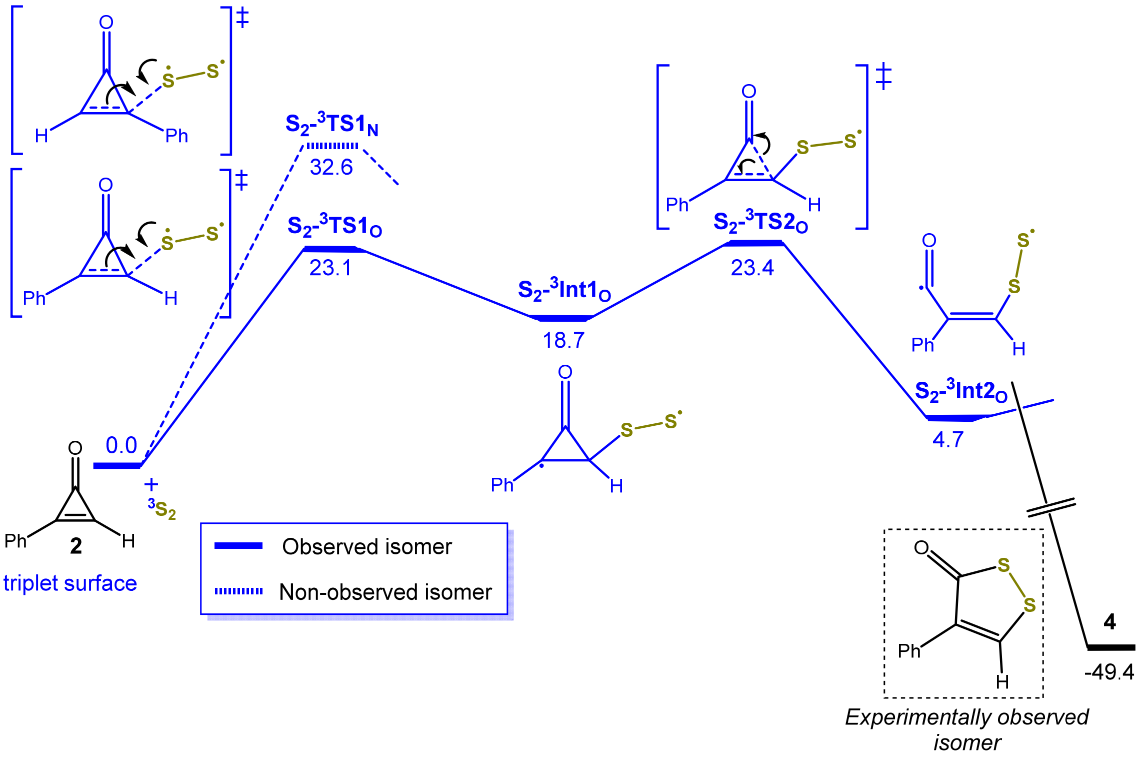

Figure S12. Free energy profile for the (3+2) cycloaddition of 2-phenylcycloprop-2-en-1-one, **2**, and triplet disulfur $^3\mathbf{S_2}$. Please note that in this profile the zero of energy is set on triplet $^3S_2$, a species 19.9 kcal·mol^{-1} less stable than $^1\mathbf{S_8}$ (see Figure S10). Dashed lines correspond to the reaction pathways leading to the experimentally non-observed regioisomer, solid ones to the observed regioisomer, **4**. Energies correspond to relative free energies in solution in kcal·mol^{-1}.

4.4. Previous postulated mechanisms

The kinetic or thermodynamic feasibility of previously postulated mechanisms in the literature was explored computationally.[13, 14] These previously postulated mechanisms are commented on the main text are presented in Figure S13 with the same notation. Here we also include another option, path d), not commented on the main text. Path d) that was not proposed for cyclopropenones but for cyclopropenethiones. All mechanisms were discarded after several explorations of the potential energy surface, the most representative results to discard them are detailed below.

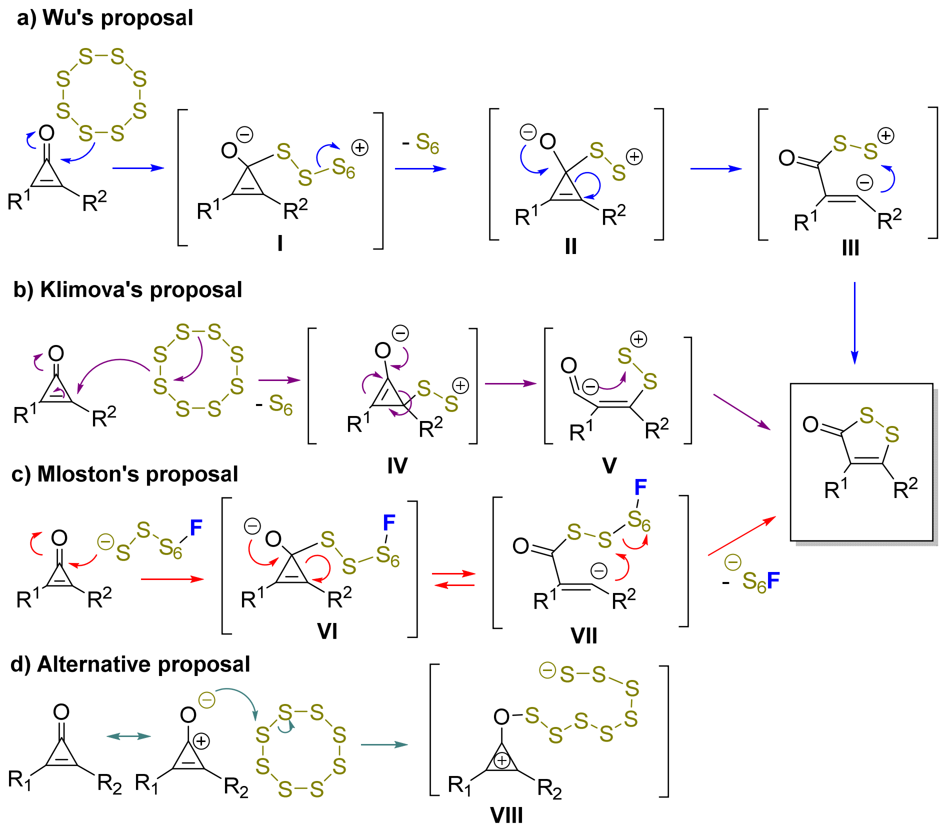

Figure S13: Previously postulated mechanisms

Mechanism a):

Transition state searches and scans on the potential energy surfaces have been performed to localize a transition state able to lead to formation of intermediate **I** or intermediate **II**. We haven't been able to find any low energy path to form any of these literature postulated intermediates.

We present in Figure S14, the results of one of the carried representative scans where we can see a continuous energy increase from cyclopropenone and S_8 separated (right), when the S-C distance between the S_8 and the carbon of the carbonyl is shortened. The scan forces the formation of the C-S bond and the energy raises up to 90.9 kcal·mol^{-1}. We can observe that

no minima or transition state appears in the scan suggesting that a direct nucleophilic addition of S_8 to the carbonyl group (Wu's proposal) is not a plausible pathway.

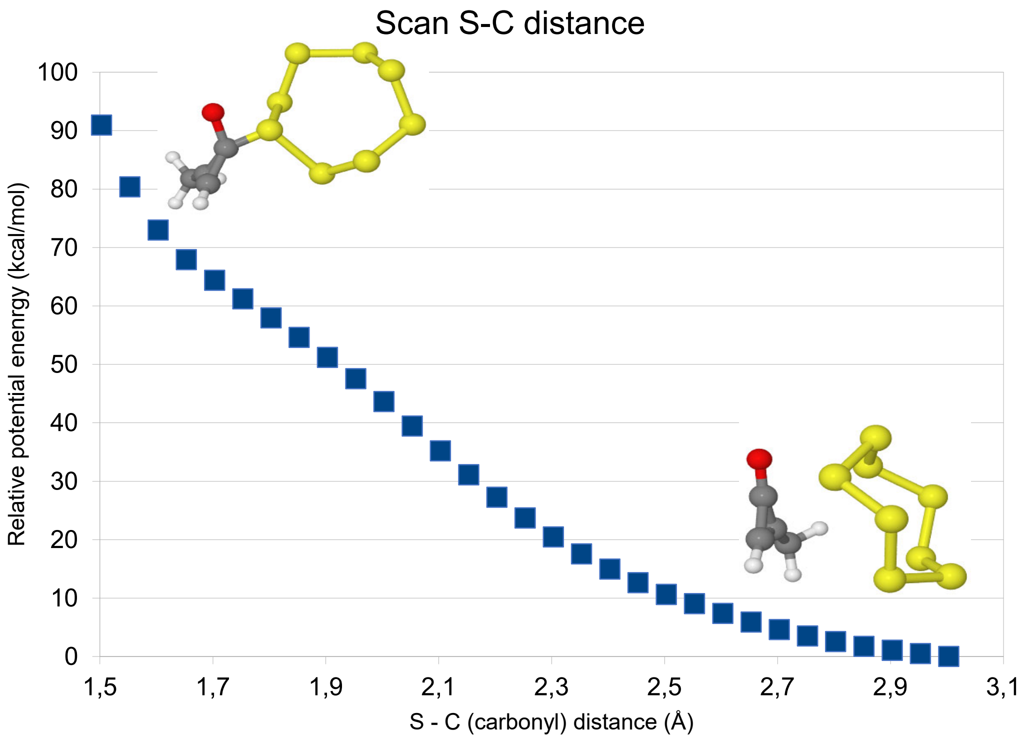

Figure S14: Scan of the S – C distance of the S_8 and the carbon of the carbonyl of the cyclopropenone on the potential energy surface. Energies correspond to potential energies at the level used for optimization, ωB97XD/6-31g(d,p)+SMD, in kcal·mol^{-1} and distances in Angstrom (Å).

Mechanism b):

Mechanism b) is similar to the one presented in section 4.2 of this supporting information. The mechanism presented in that section is the best mechanism we have been able to find for the reactivity of non-activated S_8 and cyclopropenone. The mechanism shows a high barrier of 38.9 kcal·mol^{-1}, for the concerted opening of the S_8 ring and the formation of the S-C bond. However, the scission of the sulfur chain and formation of the S_6 cycle occurs at a later stage. Attempts to find a different transition state forming the S_6 cycle at the same time as the opening of the S_8 have been unsuccessful.

Mechanism c):

This mechanism requires the addition of the ^{1}**FS**$_8^-$ anion to the carbonyl. The resulting intermediate **VI** (Scheme 2 and Figure S13) was searched but could not be located as minima, as the different attempts ended in the reactant's geometries. Scans in the potential energy

surface shortening the C-S distance between the terminal sulfur of the **$^{1}FS_8^-$** anion and the carbon of the carbonyl were performed. An increase on the energy when shorting the C-S distance was found and no indication of minima or transition state was found, see Figure S15. Also, freezing the C-S distance to 2.02 Å we optimized all other geometrical parameters and estimated its free energy in approximately 38 kcal·mol^{-1} above reactants (as this is not a stationary point on the potential energy surface the keyword freq=projected was used to minimize errors). According to these results this mechanistic possibility was also discarded.

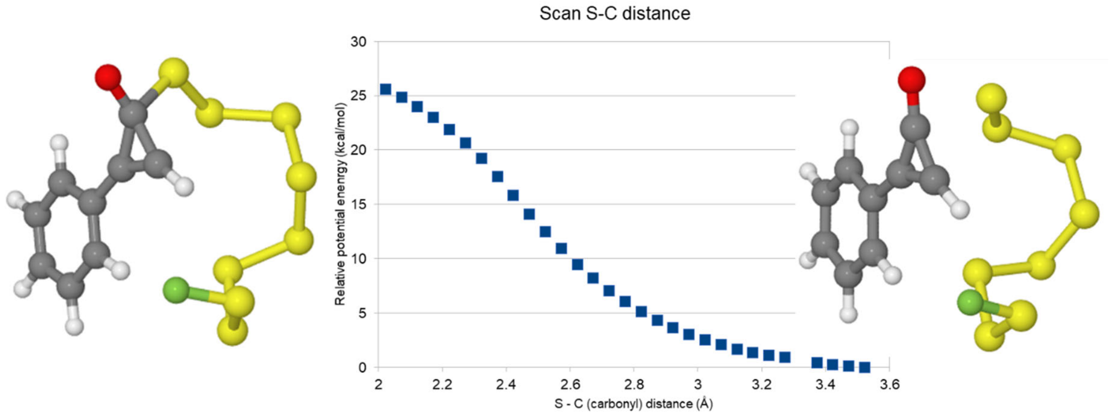

Figure S15: Scan of the S – C distance of the **$^{1}FS_8^-$** anion and the carbon of the carbonyl of the cyclopropenone on the potential energy surface. Energies correspond to potential energies at the level used for optimization, ωB97XD/6-31g(d,p)+SMD, in kcal·mol^{-1} and distances in Angstrom (Å).

Mechanism d):

Mechanism d) initially postulated for cyclopropenthiones, has been discarded on the basis that the postulated intermediate **VIII,** see Figure S16, has high energy. This literature postulated intermediate has an energy of 47.6 kcal·mol^{-1} above the reactants (S_8 and cyclopropenone), discarding this mechanistic possibility.

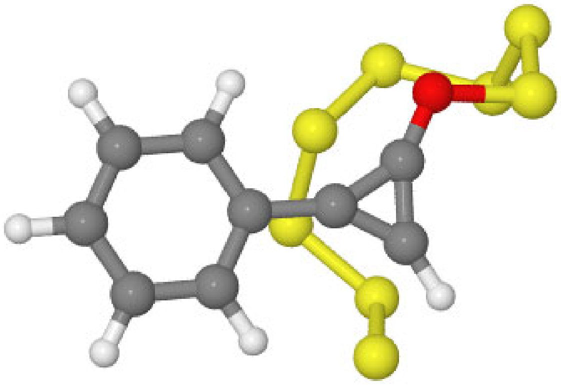

Figure S16: Geometry of literature postulated intermediate **VIII**

4.5. Reaction mechanism for the (3+2) cycloaddition of 2-phenylcycloprop-2-en-1-one, 2, with fluoroheptasulfide anion, FS_7^-.

In the main text, the key steps of the postulated mechanisms when considering ^{1}**FS**$_8^-$ as polysulfide anion were presented, see Figure 2 and Figure S24. However other polysulfide anions could also be present. In this section we consider the reactivity with ^{1}**FS**$_7^-$ for the rate and selectivity determining steps. ^{1}**FS**$_7^-$ is studied to observe the effect of shortening a bit the polysulfide chain.

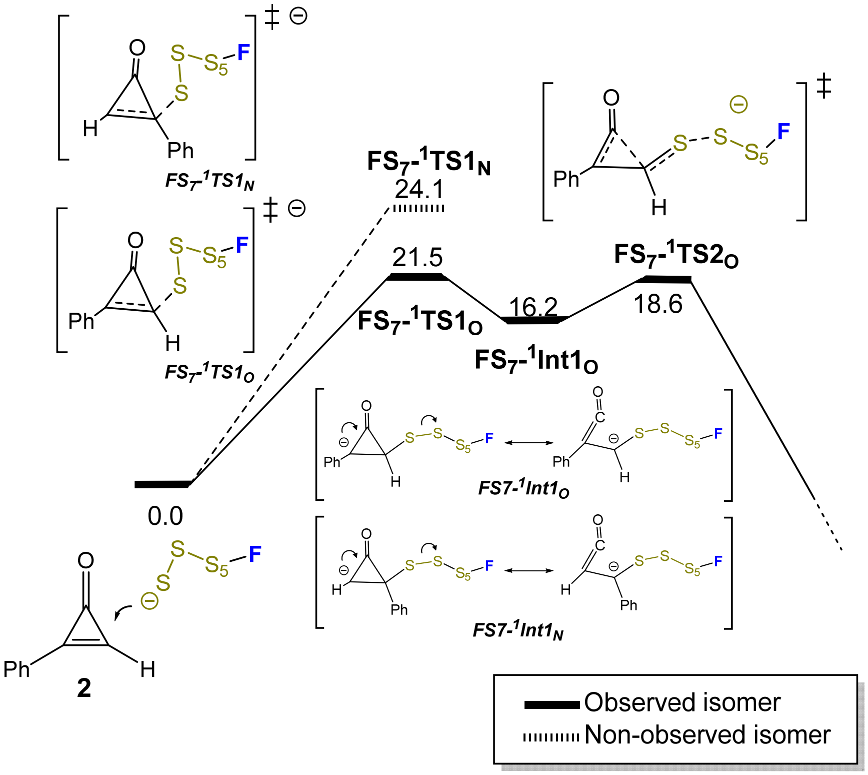

Figure S17. Free energy profile for the (3+2) cycloaddition of 2-phenylcycloprop-2-en-1-one, **2**, fluoroheptasulfide anion **FS**$_7^-$. Dashed lines correspond to the reaction pathways leading to the experimentally non-observed regioisomer, solid ones would lead to the observed regioisomer, **4**. Energies correspond to relative free energies in solution, in kcal·mol^{-1}.

4.6. Reaction mechanism for the (3+2) cycloaddition of 2-phenylcycloprop-2-en-1-one, 2, with fluorodisulfide anion, FS_2^-.

In Figure S18, the reactivity with the fluorodisulfide anion **FS_2^-** as polysulfide anion is presented. We searched the free energy profile for **FS_2^-** as we were interested to know the reactivity of the small polysulfide anions. The mechanism is similar to the one for **FS_8^-**, except for the addition of the second sulfur atom. For **FS_2^-** the **FS_2-^{1}TS3$_{OA}$** could not be located and had to be estimated as the energy of a weakly bound intermediate with a C-S distance of 3.4 Å, see Figure S19, where no transition state is observed between the two minima.

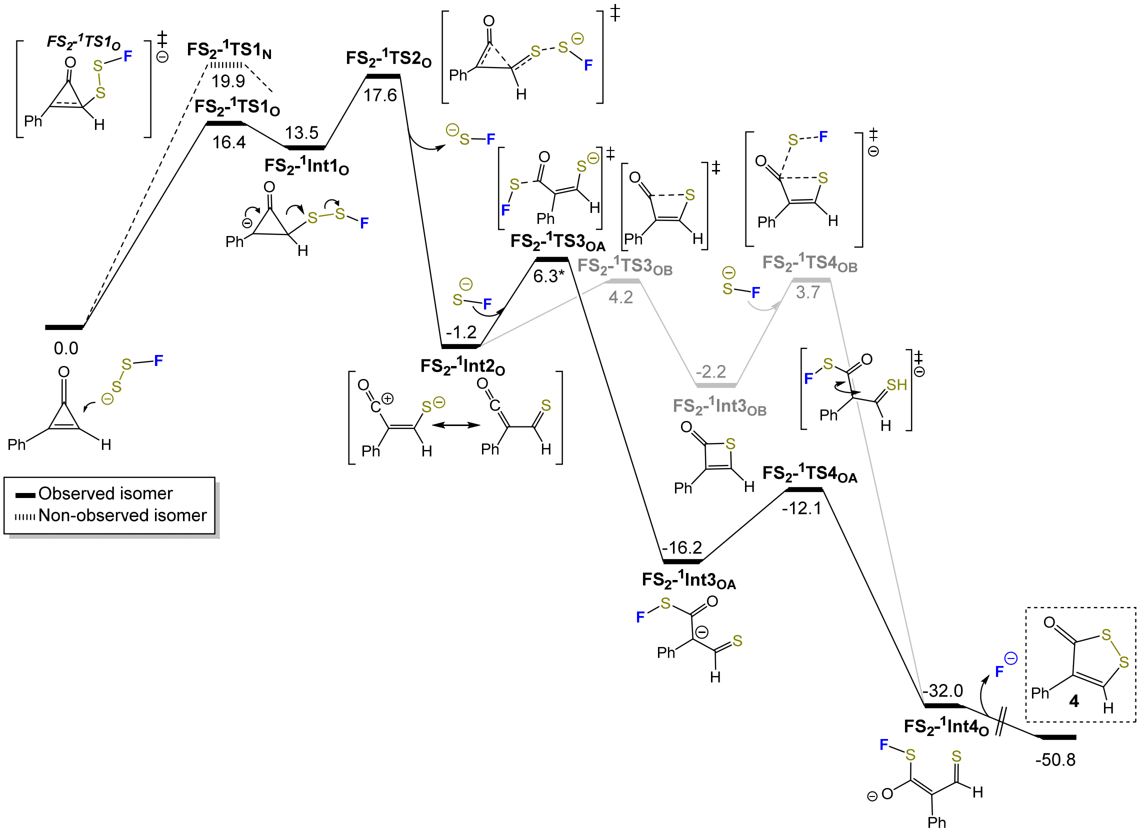

Figure S18. Free energy profile for the (3+2) cycloaddition of 2-phenylcycloprop-2-en-1-one, **2**, and fluorodisulfide anion, **FS_2^-**. Dashed lines correspond to the reaction pathways leading to the experimentally non-observed regioisomer, solid ones to the observed regioisomer, **4**. Grey solid lines indicate competing pathway leading to same products. * = No transition state could be located, energy corresponds to weakly bound intermediate with a C-S distance of 3.4 Å, see Figure S19. Energies correspond to relative free energies in solution in kcal·mol^{-1}.

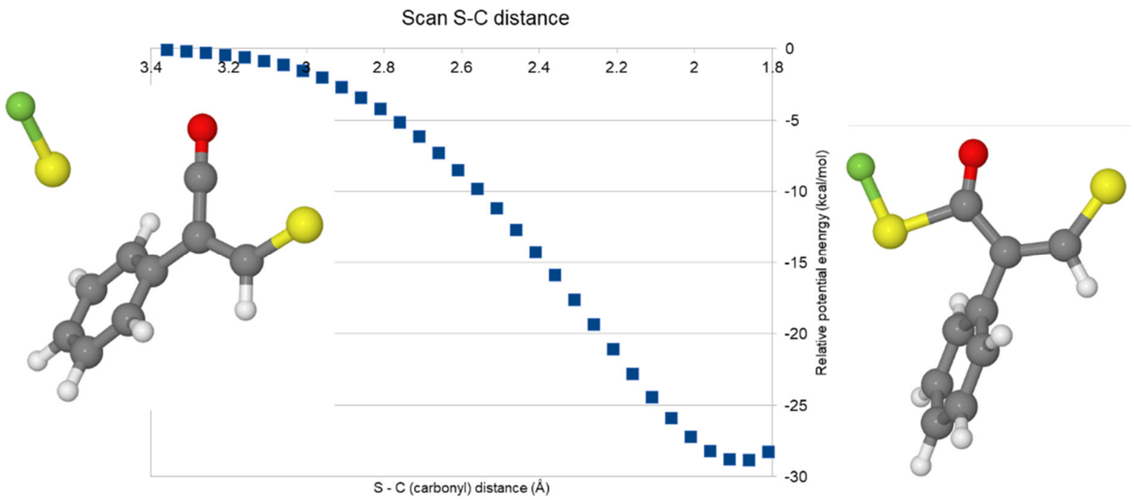

Figure S19: Scan of the S – C distance of the $\mathbf{FS_2^-}$ and the carbon of the carbonyl of the **F2-[1]Int2$_\mathbf{O}$** on the potential energy surface leading to formation of **F2-[1]Int3$_\mathbf{OA}$**. No transition state is observed between the two minima. Energies correspond to potential energies at the level used for optimization, ωB97XD/6-31g(d,p)+SMD, in kcal·mol^{-1} and distances in Angstrom (Å). Color code: S in yellow, F in green, O in red, C in grey, and H in white.

4.7. Reaction mechanism for the (3+2) cycloaddition of 2-phenylcycloprop-2-en-1-one, 2, with fluorosulfide anion, FS^-.

In the main text, the key steps of the postulated mechanisms when considering $^1FS_8^-$ as polysulfide anion were presented, see Figure 2 and Figure S24. However other polysulfide anions could also be present. In this section we consider the reactivity with the smallest fluorosulfide $^1FS^-$ for the rate and selectivity determining steps. In this case **FS-^{1}Int1$_O$** and **FS-^{1}Int1$_N$** are more stabilized than reactants.

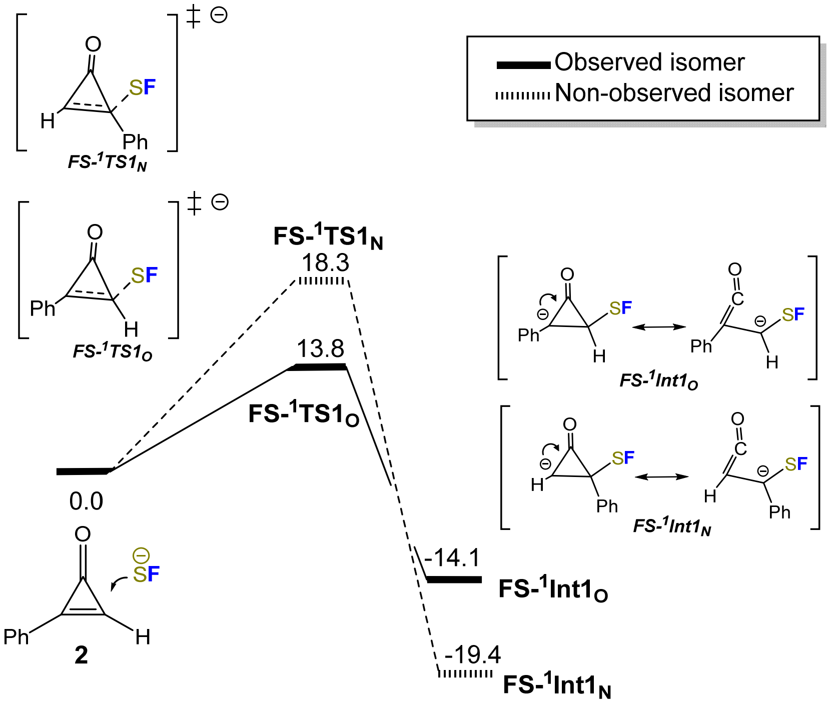

Figure S20. Free energy profile for the (3+2) cycloaddition of 2-phenylcycloprop-2-en-1-one, **2**, fluoroheptasulfide anion **FS**$^-$. Dashed lines correspond to the reaction pathways leading to the experimentally non-observed regioisomer, solid ones would lead to the observed regioisomer, **4**. Energies correspond to relative free energies in solution, in kcal·mol^{-1}**.**

4.8. Reaction mechanism for the (3+2) cycloaddition of 2-phenylcycloprop-2-en-1-one, 2, with nonasulfanide anion, HS_9^-.

In the main text, the key steps of the postulated mechanisms when considering **$^1FS_8^-$** as polysulfide anion were presented, see Figure 2 and Figure S24. However other polysulfide anions could also be present. In this section we consider the reactivity with **$^1HS_9^-$** for the rate and selectivity determining steps. We considered **$^1HS_9^-$** as it could be formed from **HS^-** and **$^1S_8$**.

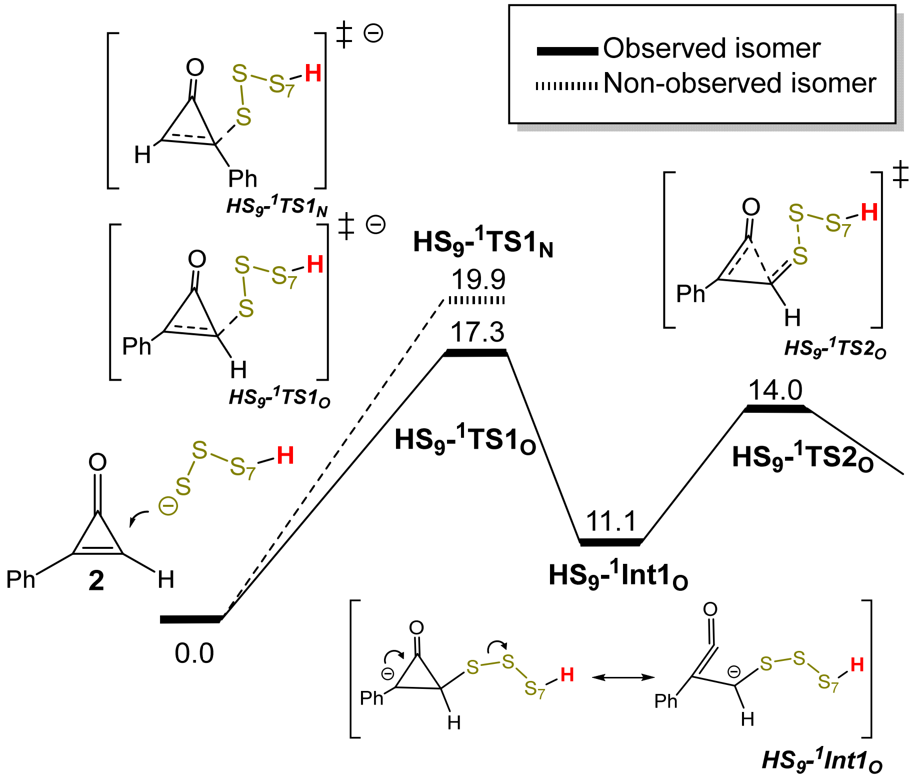

Figure S21. Free energy profile for the (3+2) cycloaddition of 2-phenylcycloprop-2-en-1-one, **2**, and fluorodisulfide anion, **HS_9^-**. Dashed lines correspond to the reaction pathways leading to the experimentally non-observed regioisomer, solid ones to ones leading to the observed regioisomer, **4**. Energies correspond to relative free energies in solution in kcal·mol^{-1}.

4.9. Reaction mechanism for the (3+2) cycloaddition of 2-phenylcycloprop-2-en-1-one, 2, with trisulfanide anion, HS_3^-.

In Figure S22, the reactivity with the trisulfanide anion $\mathbf{HS_3^-}$ as polysulfide is presented. $\mathbf{HS_3^-}$ has been studied as example of short polysulfide. It could be formed from $\mathbf{HS_9^-}$ leading to $\mathbf{^1S_6}$ and $\mathbf{HS_3^-}$. The mechanism is similar to the one for $\mathbf{FS_2^-}$, and as in that case (see above) the $\mathbf{HS_3\text{-}^1TS3_{OA}}$ could not be located and the energy has been estimated to that of a weakly bound intermediate with a C-S distance of 3.4 Å.

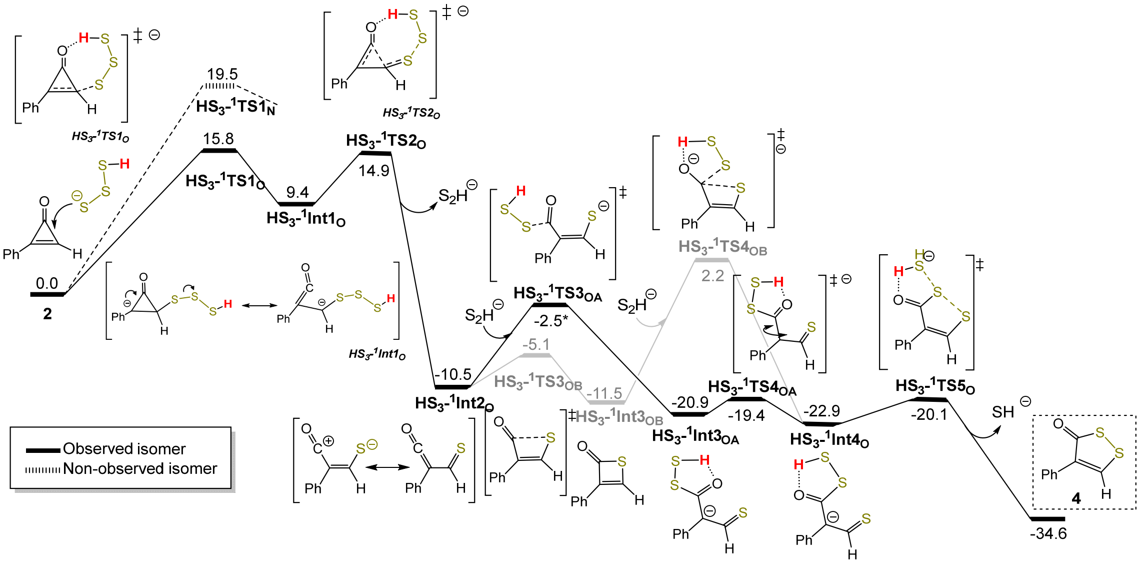

Figure S22. Free energy profile for the (3+2) cycloaddition of 2-phenylcycloprop-2-en-1-one, **2**, and $\mathbf{HS_3^-}$. Dashed lines correspond to the reaction pathways leading to the experimentally non-observed regioisomer, solid ones to the observed regioisomer, **4**. * = No transition state could be located, energy corresponds to weakly bound intermediate with a C-S distance of 3.4 Å. Energies correspond to relative free energies in solution in kcal·mol^{-1}.

4.10. Reaction mechanism for the (3+2) cycloaddition of 2-phenylcycloprop-2-en-1-one, 2, with nonasulfanediide anion, S_9^-.

In Figure S23, the reactivity with the nonasulfanediide $\mathbf{S_9}^-$ as polysulfide is presented. The mechanism is similar to the one for FS_8^-. We have considered $\mathbf{S_9}^-$ as it could be formed from the reaction of $^1\mathbf{S_8}$ and $\mathbf{S}^{2-}$ impurities.

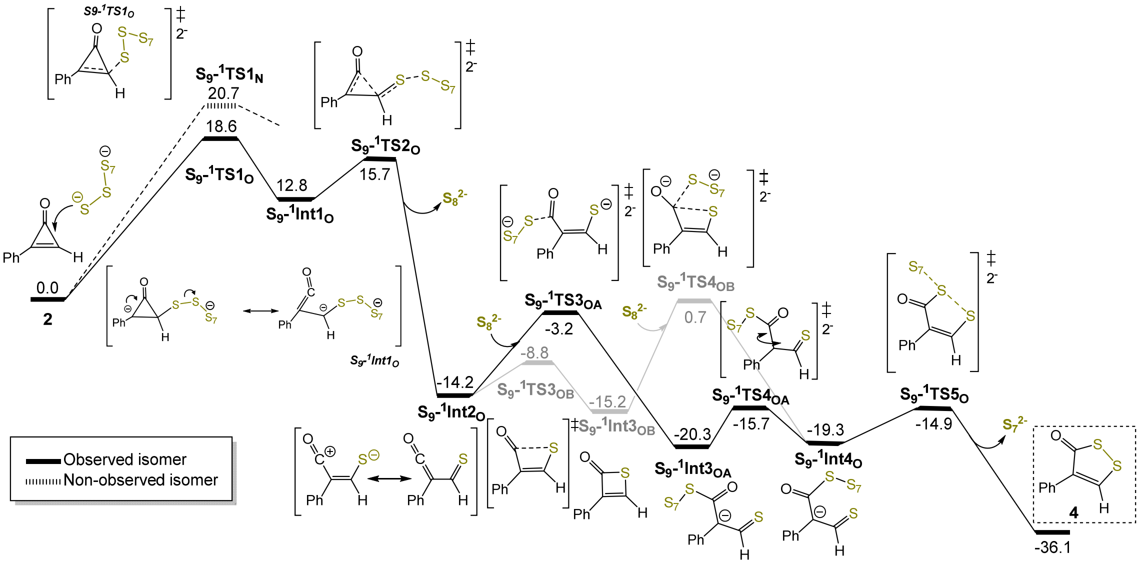

Figure S23. Free energy profile for the (3+2) cycloaddition of 2-phenylcycloprop-2-en-1-one, **2**, and $^1\mathbf{S_9}^-$. Dashed lines correspond to the reaction pathways leading to the experimentally non-observed regioisomer, solid ones to the observed regioisomer, **4**. Energies correspond to relative free energies in solution in kcal·mol^{-1}.

4.11. More details on the reaction mechanism for the (3+2) cycloaddition of 2-phenylcycloprop-2-en-1-one, 2, with fluorooctasulfide anion, FS_8^-.

As mentioned in the main text, ^{1}**FS$_8^-$** anion adds to the less hindered carbon of the cyclopropenone's C-C double bond, via **FS8-^{1}TS1$_O$**, forming **FS8-^{1}Int1$_O$**, see Figure S24. The equivalent transition state leading to the non-observed product **FS8-^{1}TS1$_N$**, is 3.0 kcal·mol^{-1} higher in energy and hence the formation of the non-observed product is disfavoured. Ring opening of the enolate **FS8-^{1}Int1$_O$**, leads to ketenethialdehyde intermediate **FS8-^{1}Int2$_O$**. The first step is the selectivity and rate-determining step (barrier of 23.2 kcal·mol^{-1}). Ketenethialdehyde **FS8-^{1}Int2$_O$** can form thiet-2-one **FS8-^{1}Int3$_{OB}$**.

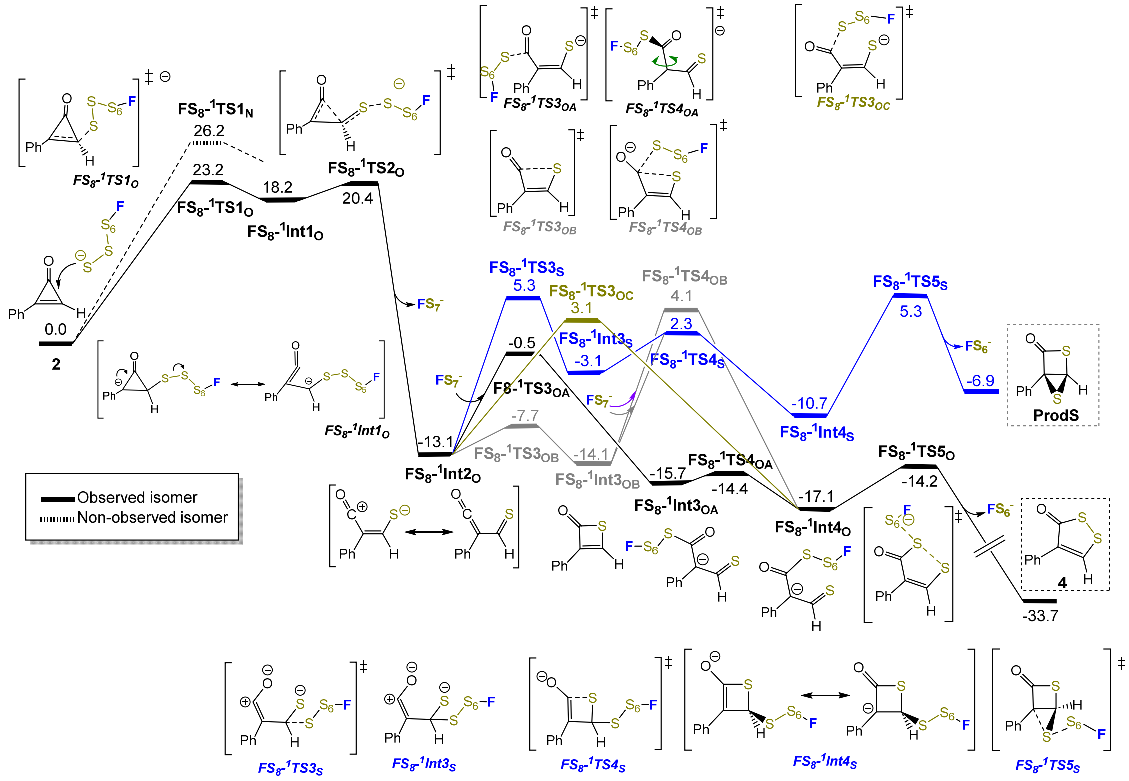

Figure S24. Free energy profile for the (3+2) cycloaddition of 2-phenylcycloprop-2-en-1-one, **2**, fluorooctasulfide anion **FS$_8^-$**. Dashed lines correspond to the reaction pathways leading to the experimentally non-observed regioisomer, solid ones to the observed regioisomer, **4**. Grey and brown solid lines indicate competing pathways leading to same products. Energies correspond to relative free energies in solution, in kcal·mol^{-1}**.**

Nucleophilic attack to the carbonyl group of the open **FS8-^{1}Int2$_O$** and the cyclic thiet-2-one **FS8-^{1}Int3$_{OB}$** can lead to **FS8-^{1}Int4$_O$** thioenolate. The lowest energy path A, black in Figure S24, consists on the nucleophilic attack to the open **FS8-^{1}Int2$_O$** leading to **FS8-^{1}Int3$_{OA}$** (barrier 12.6 kcal·mol^{-1} from **FS8-^{1}Int2$_O$**, 13.6 kcal·mol^{-1} from **FS8-^{1}Int3$_{OB}$**). A low barrier C-C bond rotation leads to **FS8-^{1}Int$_{4O}$**. On the other hand, the nucleophilic attack can take place to cyclic thiet-2-one **FS8-^{1}Int3$_{OB}$** the C-S bond is formed concertedly with the ring opening, see path B in grey (barrier of 18.2 kcal·mol^{-1}). Another path can connect **FS8-^{1}Int2$_O$** and **FS8-^{1}Int4$_O$** in a single step through **FS8-^{1}TS3$_{OC}$**, path C in brown, is similar to A but more energy demanding (3.6

kcal·mol^{-1} higher). Thioenolate **FS8-^{1}Int4$_O$** finally can undergo nucleophilic ring closing and fluorohexasulfide displacement to form the experimentally obtained regiosiomer **4**.

However, it is interesting to note that both the open ketenethialdehyde **FS8-^{1}Int2$_O$** can also lead to the formation of an episulfide 2 -thietanone, ProdS, instead of the 1,2-dithiolan-3-one experimental product **4**. The transition state are a bit higher in energy than the preferred black pathway (4.8 above **FS8-^{1}TS3$_{OA}$**) and can be discarded, see blue pathway in Figure S24. Moreover, as **ProdS** is thermodynamically less favoured than the key intermediates and product **4**, even if a small amount of **ProdS** was formed it is expected to revert towards productive intermediates and finally evolve to product **4**.

5. Optimized Geometries and Energies of Relevant Species

Cartesian coordinates (in Angstrom) and potential energies (in Hartree)

More information extracted in the outputs is available at ioChem-BD, https://iochem-bd.urv.es/browse/

2

E= -421.584249 h.

C	2.658842	-0.129349	-0.017385
C	2.413420	1.309755	0.132660
C	1.290165	0.404832	-0.035083
O	3.454054	-1.102698	-0.079033
H	2.788671	2.302253	0.270631
C	-0.230109	0.159693	-0.018579
C	-1.115404	1.245187	-0.061939
C	-0.728265	-1.148876	0.039798
C	-2.498853	1.022110	-0.046920
H	-0.735051	2.244309	-0.106511
C	-2.111715	-1.371953	0.054816
H	-0.052323	-1.977675	0.072906
C	-2.997009	-0.286459	0.011457
H	-3.174795	1.850909	-0.080026
H	-2.492067	-2.371075	0.099392
H	-4.053303	-0.456783	0.022924

S_8

E=-3185.37466915 h.

S	-1.868460	1.468951	-0.499296
S	-2.360106	-0.282761	0.499117
S	-1.468831	-1.868429	-0.499405
S	0.282424	-2.359771	0.499459
S	1.868828	-1.468842	-0.499262
S	2.360084	0.282611	0.499196
S	1.468791	1.868581	-0.499339
S	-0.282731	2.359660	0.499529

S_2

E= -796.279781 h.

S	0.000000	0.000000	0.888800
S	0.000000	0.000000	-0.888800

$^3S_2$

E= -796.314858 h.

S	0.000000	0.000000	0.888800
S	0.000000	0.000000	-0.888800

S_6F^-

E= -2488.949070 h.

S	-1.095992	2.055227	0.504750
S	1.786254	0.917774	-0.107110
S	1.355758	-0.802558	0.877922
S	0.052015	-1.900486	-0.353962
S	-1.842841	-1.225083	0.191329
S	-2.088048	0.667687	-0.664300
F	3.258406	0.511002	-0.797562

S_8F^-

E= -3285.299120 h.

S	1.210999	2.171212	-0.515498
S	-0.556810	2.407251	0.535155
S	-1.961076	0.934335	-0.359041
S	-1.631444	-0.552183	1.061124
S	-0.907885	-2.054504	-0.184634
S	1.000535	-2.363257	0.367681
S	2.238604	-1.044589	-0.680411
S	2.419268	0.726228	0.392507
F	-3.221671	-0.399097	-1.096681

^{3}lin-S_8

E= -3185.321056 h.

S	-2.169777	1.242210	0.729208
S	-0.968088	2.543934	-0.226521
S	-2.500683	-0.389350	-0.562659
S	-1.409016	-1.993885	0.145454
S	0.482813	-1.947607	-0.741805
S	1.836050	-1.455465	0.732604
S	1.978524	0.665007	0.798695
S	2.750177	1.335157	-0.874976

TS (from ^{3}lin-S_8 to $^3S_2$ + S_6)

E= -3185.303100 h.

S	2.563802	0.593774	-0.975407
S	1.740401	1.890003	0.316600
S	2.535691	-1.280010	-0.007213
S	0.544638	-1.840629	-0.153633
S	-0.423936	-0.683470	1.299532
S	-0.689175	1.150508	0.430784
S	-2.880045	1.008346	-0.369413
S	-3.391376	-0.838522	-0.541250

FS8-^{1}Int1$_O$

E= -3706.865001 h.

S	-0.480868	-1.891118	-1.099896
S	1.133713	-3.129892	-1.303884
S	-1.223876	-2.043639	0.850707
S	-2.371109	-3.777736	0.973582
S	-4.317497	-3.300396	0.422895
S	-4.493781	-3.635216	-1.614655
S	-4.043064	-1.804054	-2.556581
S	-5.754744	-0.777202	-2.740265

F	-5.855442	0.065847	-1.329249
C	2.766822	-1.868380	-0.314224
C	3.568413	-2.556794	0.695043
C	4.097367	-2.253176	-0.542692
O	3.628450	-3.018989	1.832261
C	5.163907	-2.206846	-1.519595
C	4.936307	-1.648016	-2.787023
C	6.435889	-2.723934	-1.225892
C	5.954645	-1.607512	-3.733703
H	3.953403	-1.248715	-3.022811
C	7.452089	-2.675734	-2.173904
H	6.620335	-3.161528	-0.248793
C	7.216432	-2.119020	-3.431282
H	5.763921	-1.174151	-4.711307
H	8.432322	-3.076601	-1.932462
H	8.010912	-2.085292	-4.170648
H	2.194576	-0.948728	-0.425466

FS8-[1]Int2$_{O}$

Eopt -819.789238

C	-1.692470	-0.919777	-0.375457
C	-1.251983	1.239707	0.464270
O	-1.674416	2.242538	0.849001
C	-0.744750	0.076506	0.019856
S	-3.333885	-0.794483	-0.309371
C	0.745540	-0.034816	-0.016670
C	1.392547	-0.998714	0.763255
C	1.497298	0.829500	-0.816098
C	2.778867	-1.102981	0.728604
H	0.808980	-1.661522	1.394156
C	2.886594	0.728694	-0.838967
H	0.993179	1.576907	-1.422357
C	3.527758	-0.238956	-0.070339
H	3.276624	-1.855165	1.332727
H	3.464907	1.404522	-1.461198
H	4.610224	-0.320025	-0.090860
H	-1.252830	-1.848269	-0.745269

FS8-[1]Int3$_{OA}$

E= -3706.94089715 h.

6	1.469190	4.124572	0.384018
6	2.033359	3.457364	1.469156
6	2.520713	2.161347	1.316062
6	2.441032	1.504681	0.080280
6	1.877167	2.191794	-1.003491
6	1.397575	3.490466	-0.855487
6	2.963278	0.113195	-0.091696
6	2.247934	-0.979741	0.501358
8	2.476726	-2.161377	0.532383
6	4.089186	-0.072004	-0.887432
16	4.961995	-1.447883	-1.347129
16	0.592802	-0.371108	1.293857
16	-0.272084	-2.158813	1.803738
16	-0.964431	-3.017322	0.037260
16	-2.816179	-2.177235	-0.394198
16	-2.482069	-0.606981	-1.707117
16	-2.266227	1.135952	-0.540850
16	-4.084659	1.953105	-0.335573
9	-4.684233	1.231153	1.018644
1	2.971192	1.648968	2.161945
1	1.809310	1.693752	-1.966978
1	2.101781	3.948718	2.435282
1	0.961137	4.005248	-1.706478
1	1.090240	5.135405	0.502165
1	4.467649	0.873087	-1.289209

FS8-[1]Int3$_{OB}$

E= -819.790793 h.

C	1.588438	-1.337862	0.060128
C	1.867270	0.779810	0.025395
O	1.876509	1.893799	0.080710
C	0.750784	-0.259748	0.023967
S	3.140492	-0.442084	-0.070523
C	-0.784175	-0.136319	0.007598
C	-1.580066	-1.289703	0.021153
C	-1.385095	1.129385	-0.020854
C	-2.976878	-1.177383	0.006258
H	-1.121250	-2.256097	0.042877
C	-2.781907	1.241705	-0.035748
H	-0.777414	2.010019	-0.031207
C	-3.577799	0.088321	-0.022190
H	-3.584559	-2.058017	0.016608
H	-3.240723	2.208098	-0.057471
H	-4.644296	0.174080	-0.033563
H	1.384863	-2.384361	0.151209

FS8-[1]Int4$_{O}$

E= -3706.947023 h.

C	2.488784	1.918085	-0.263600
C	1.869526	-0.304447	0.549172
O	1.908185	-1.456157	0.828498
C	2.825912	0.618727	0.012721
S	0.029810	0.939292	0.796085
S	0.975350	2.643029	-0.013444
C	4.208977	0.115475	-0.225475
C	5.310536	0.916136	0.104336
C	4.442989	-1.142267	-0.796378
C	6.608592	0.479473	-0.145558
H	5.149664	1.883061	0.572868
C	5.741558	-1.581000	-1.036846
H	3.605515	-1.779724	-1.059373
C	6.829833	-0.771785	-0.716035
H	7.448377	1.115669	0.117869
H	5.902334	-2.557728	-1.483592
H	7.842134	-1.115964	-0.904914
S	-0.756598	-0.773319	1.699759
S	-0.993803	-2.170538	0.196777
S	-3.019342	-2.555074	-0.070968
S	-3.853485	-1.139990	-1.349441
S	-4.796825	0.277121	-0.102417

S	-3.552023	1.817832	0.192291
F	-3.884294	2.827218	-1.071241
H	3.255555	2.554944	-0.701934

FS8-^{1}TS1$_N$

E= -3706.858564 h.

C	-3.315664	0.126749	0.866645
C	-3.060022	1.239369	1.785244
O	-3.128155	2.451319	1.982937
C	-2.785778	-0.055074	2.156421
S	-0.102540	-0.242105	0.286685
S	-1.793618	-0.032230	-0.816325
S	0.652790	1.644909	0.798440
S	1.745255	2.355828	-0.824884
S	3.698621	1.668983	-0.639630
S	3.834341	-0.125770	-1.667103
S	3.426959	-1.645248	-0.264138
S	5.166286	-2.177817	0.578029
F	5.311040	-1.149233	1.855914
C	-4.475641	-0.427206	0.130755
C	-5.123440	0.332874	-0.850040
C	-4.960715	-1.704398	0.430915
C	-6.230926	-0.177963	-1.521313
H	-4.755529	1.328512	-1.082636
C	-6.068317	-2.214872	-0.240425
H	-4.466539	-2.294978	1.197880
C	-6.705642	-1.453697	-1.219537
H	-6.727319	0.422295	-2.278257
H	-6.435886	-3.207982	0.001374
H	-7.570162	-1.851645	-1.742519
H	-2.511288	-0.803150	2.888246

FS8-^{1}TS1$_O$

E= -3706.87341629 h.

C	-1.262628	1.289523	2.089271
C	-0.542498	0.062198	1.885137
C	0.376607	-0.691268	2.253635
C	-1.643693	0.360352	1.114252
S	0.117487	2.683837	-0.632730
S	-0.378015	3.300892	1.250988
S	2.202390	2.701948	-0.805970
S	2.974292	1.049365	0.188283
S	3.283513	-0.466564	-1.198519
S	1.588030	-1.628663	-1.396441
S	1.653186	-2.921688	0.289700
S	-0.154074	-2.781019	1.164746
F	-0.913909	-4.061352	0.358654
H	-1.748546	1.829336	2.893394
C	-2.675324	0.134900	0.118385
C	-3.581504	1.175424	-0.140875
C	-2.794948	-1.063558	-0.600917
C	-4.578214	1.024472	-1.098462
H	-3.490206	2.105622	0.412850
C	-3.797816	-1.210419	-1.553378
H	-2.115393	-1.885581	-0.405666
C	-4.690242	-0.169871	-1.808738
H	-5.269591	1.839742	-1.290012
H	-3.882754	-2.144575	-2.100635
H	-5.468895	-0.290092	-2.555851

FS8-^{1}TS2$_O$

E= -3706.86900773 h.

C	3.089065	-1.748041	-0.015180
C	4.570422	-1.720263	-1.077966
O	5.155473	-2.492839	-1.793211
C	4.112466	-0.722637	-0.353895
S	0.194451	-0.330601	-0.213869
S	1.569295	-1.882119	-0.833893
C	4.419249	0.628637	0.068597
C	3.604663	1.277335	1.010426
C	5.532953	1.322748	-0.438061
C	3.891415	2.572844	1.428921
H	2.739371	0.752516	1.406597
C	5.814601	2.617279	-0.016516
H	6.175992	0.836866	-1.167572
C	4.996986	3.252940	0.919228
1	3.245763	3.055167	2.157961
H	6.679657	3.135196	-0.421978
H	5.218843	4.264644	1.245121
S	-0.576638	-0.860365	1.638903
S	-2.108151	-2.236917	1.333618
S	-3.878335	-1.173140	1.096001
S	-4.124957	-0.809969	-0.929184
S	-3.220988	1.056453	-1.307694
S	-4.613232	2.473050	-1.038105
F	-4.497568	2.831153	0.565056
H	3.120672	-2.249384	0.954270

FS8-^{1}TS3$_{OA}$

E= -3706.91596015 h.

6	1.454344	4.199528	0.579616
6	2.337782	3.473986	1.377140
6	2.707514	2.181932	1.018179
6	2.181270	1.589557	-0.135619
6	1.291955	2.321043	-0.929778
6	0.938049	3.620676	-0.576923
6	2.572533	0.215784	-0.536830
6	2.802187	-0.773658	0.371640
8	3.178931	-1.690065	0.958115
6	2.785246	-0.166305	-1.891103
16	3.182769	-1.651254	-.515301
16	0.547513	-.160032	2.330739
16	-0.153722	-.094215	2.335137
16	-0.200273	-.818053	0.381734
16	-1.980982	-2.231012	-.545527
16	-1.626147	-0.427557	-.505636
16	-2.238871	1.086255	-.171711
16	-4.139607	1.532101	-.625517
9	-5.023963	0.476823	0.285492
1	3.411548	1.631748	1.635837

1	0.862062	1.870277	-1.819498
1	2.750185	3.918356	2.277882
1	0.245037	4.174763	-1.202804
1	1.170897	5.209772	0.858358
1	2.668356	0.669203	-2.584714

FS8-[1]TS3$_{OB}$

E= -819.780366 h.

C	1.775131	-1.229202	0.000000
C	1.619609	1.056903	-0.000001
O	1.394546	2.333516	-0.000004
C	0.786535	-0.025172	-0.000001
S	3.312664	-0.601550	0.000002
C	-0.752404	-0.082309	-0.000002
C	-1.407594	-1.321119	-0.000002
C	-1.497650	1.104506	0.000002
C	-2.808029	-1.373113	-0.000002
H	-0.838582	-2.227278	-0.000003
C	-2.898086	1.052512	0.000004
H	-0.997399	2.050365	0.000006
C	-3.553275	-0.186297	0.000000
H	-3.308280	-2.318971	-0.000002
H	-3.467097	1.958672	0.000007
H	-4.622538	-0.225996	-0.000000
H	1.489483	-2.260369	0.000003

FS8-[1]TS4$_{OA}$

E= -3706.93419450 h.

6	6.740871	-0.339981	-1.083768
6	6.162020	0.826829	-0.589663
6	4.876900	0.808093	-0.054256
6	4.132598	-0.381414	-0.004991
6	4.721310	-1.545257	-0.527904
6	6.011635	-1.527032	-1.048062
6	2.763420	-0.437510	0.574460
6	1.895098	0.725997	0.310690
8	2.094977	1.883260	0.574694
6	2.348975	-1.501047	1.334125
16	0.887266	-1.761797	2.195678
16	0.416799	0.241995	-0.701354
16	-0.469323	2.051028	-1.107997
16	-1.600007	2.581139	0.558464
16	-3.496741	1.765748	0.323069
16	-3.527998	-0.039851	1.337533
16	-2.938098	-1.516282	-0.045931
16	-4.584326	-2.131951	-1.009920
9	-4.699532	-1.094547	-2.284499
1	4.447795	1.722540	0.340509
1	4.153811	-2.471377	-0.541830
1	6.716966	1.760558	-0.611064
1	6.441536	-2.443081	-1.443314
1	7.744747	-0.323728	-1.497184
1	3.112994	-2.272600	1.459373

FS8-[1]TS4$_{OB}$

E= -3706.908655 h.

C	3.766355	0.800623	1.146411
C	2.874370	-0.695684	-0.171828
O	2.855107	-1.302366	-1.203792
C	2.808255	0.753416	0.199486
S	1.032836	-1.459722	1.164212
S	4.370582	-0.838618	1.267723
C	1.919302	1.761654	-0.376425
C	1.836961	3.049014	0.177240
C	1.125815	1.456460	-1.491126
C	0.991838	4.003653	-0.376212
H	2.434698	3.300134	1.048981
C	0.279642	2.415786	-2.040789
H	1.175243	0.462371	-1.923358
C	0.208941	3.692621	-1.488475
H	0.940384	4.994679	0.065141
H	-0.325875	2.162055	-2.905785
H	-0.452196	4.439264	-1.917803
S	0.345414	-2.940470	-0.037420
S	-0.655215	-2.173460	-1.707267
S	-2.616649	-1.716249	-1.126873
S	-2.822825	0.340696	-1.180121
S	-2.083778	1.134065	0.623697
S	-3.545932	1.031873	1.990827
F	-3.332480	-0.450633	2.682528
H	4.119406	1.640996	1.734254

FS8-[1]TS5$_{O}$

E= -3706.943035 h.

C	2.189724	-0.083311	1.972328
C	1.021941	-1.007333	0.236875
O	0.949043	-1.403737	-0.907042
C	2.251709	-0.339942	0.618279
S	-0.339378	-1.412374	1.443020
S	0.972005	-0.434787	3.102387
C	3.378288	-0.031983	-0.311879
C	4.697632	-0.339131	0.044862
C	3.158643	0.610961	-1.538917
C	5.764423	-0.003075	-0.786804
H	4.889574	-0.856000	0.981432
C	4.221322	0.940240	-2.373499
H	2.144886	0.858144	-1.837603
C	5.530894	0.636930	-2.000766
H	6.778344	-0.252067	-0.486948
H	4.027160	1.443055	-3.316701
H	6.359884	0.895897	-2.652730
S	-1.623188	-2.683107	-0.065389
S	-2.213342	-1.549888	-1.694826
S	-3.962017	-0.551361	-1.133927
S	-3.501116	1.462340	-1.017505
S	-2.689569	1.841067	0.888562
S	-0.687485	1.833105	0.696624
F	-0.382421	3.369845	0.170594
H	3.067559	0.432192	2.367536

4, Prod$_{O}$

E= -1218.000185 h.

C	1.006305	-1.353290	0.000137
C	1.043203	1.067839	-0.000102
O	0.609118	2.194685	-0.000181
C	0.322835	-0.191997	0.000002
S	2.858473	0.794318	-0.000104
S	2.758190	-1.277232	0.000144
C	-1.216632	-0.151500	0.000002
C	-1.882138	1.074703	-0.000141
C	-1.945661	-1.340645	0.000033
C	-3.276360	1.111657	0.000429
H	-1.307098	2.012023	0.000611
C	-3.340313	-1.303853	-0.000392
H	-1.421299	-2.307172	-0.000034
C	-4.005728	-0.077981	-0.000056
H	-3.800990	2.078125	0.000951
H	-3.914857	-2.241602	-0.000782
H	-5.105015	-0.048594	0.000335
H	0.576642	-2.333234	0.000248

FS8-^{1}TS3$_{S}$

E= -3706.90532661 h.

6	-2.385615	2.073686	-0.698495
6	-2.891501	1.247353	0.388942
16	-0.229606	1.077950	-1.256546
16	-1.933747	3.697570	-0.472018
6	-3.415908	-0.132739	0.214260
6	-3.140868	-1.134351	1.153093
6	-4.187989	-0.453046	-0.909589
6	-3.629466	-2.425681	0.973989
1	-2.525971	-0.909979	2.020422
6	-4.655807	-1.750171	-1.095991
1	-4.430658	0.313291	-1.639643
6	-4.381494	-2.741087	-0.155099
1	-3.406389	-3.189489	1.712816
1	-5.246636	-1.983935	-1.976589
1	-4.751786	-3.751160	-0.300824
16	-0.112080	-0.929075	-1.708130
16	0.171417	-2.025023	0.047515
16	2.221752	-2.177122	0.393646
16	2.774102	-0.578747	1.593879
16	3.359591	0.970275	0.288840
16	5.340802	0.814028	0.025326
9	5.486950	-0.210280	-1.259025
1	-2.719876	1.756620	-1.681930
6	-2.831045	1.760298	1.620547
8	-2.832343	2.174239	2.705853

FS8-^{1}TS4$_{S}$

E= -3706.91066875 h.

6	2.739309	-1.208263	-0.223938
6	2.932331	0.154494	-0.058070
16	0.584348	-1.909575	0.958105
16	4.059531	-1.822877	0.814317
6	2.239142	1.310742	-0.607469
6	2.618457	2.616721	-0.253589
6	1.170698	1.144561	-1.503872
6	1.953963	3.716169	-0.785843
1	3.438684	2.762196	0.442265
6	0.513260	2.249531	-2.036065
1	0.852618	0.143986	-1.782976
6	0.899248	3.541133	-1.681089
1	2.262916	4.717419	-0.499152
1	-0.306764	2.098235	-2.731924
1	0.384135	4.401359	-2.097626
16	-0.429925	-3.111196	-0.341369
16	-1.350291	-2.009502	-1.863381
16	-3.116084	-1.183831	-1.087584
16	-2.855332	0.864361	-1.000059
16	-1.856559	1.337313	0.791014
16	-3.235483	1.529614	2.233298
9	-3.379649	0.004978	2.846094
1	2.313634	-1.758960	-1.051928
6	4.031981	0.097392	0.872044
8	4.759752	0.843396	1.463047

FS8-^{1}Int4$_{S}$

E= -3706.92927213 h.

6	2.196545	-1.289738	-0.103223
6	2.880172	0.021057	0.093907
16	0.643014	-1.307496	0.898554
16	3.591990	-2.249071	0.607547
6	2.467151	1.333980	-0.346669
6	3.213620	2.487883	-0.020065
6	1.309547	1.514087	-1.133990
6	2.815169	3.746499	-0.453055
1	4.113305	2.374673	0.577079
6	0.915094	2.779510	-1.557357
1	0.716307	0.649805	-1.420519
6	1.660815	3.908810	-1.221704
1	3.412748	4.614132	-0.184600
1	0.017648	2.881485	-2.162758
1	1.351945	4.894950	-1.554853
16	-0.417606	-2.957815	0.263949
16	-1.340355	-2.452906	-1.541363
16	-3.068753	-1.384191	-1.106515
16	-2.611358	0.628796	-1.289786
16	-2.043684	1.311708	0.621502
16	-3.710433	1.902997	1.564291
9	-4.249362	0.530804	2.300549
1	1.937085	-1.598465	-1.120997
6	4.072408	-0.377859	0.706022
8	5.103383	0.098559	1.135954

FS8-^{1}TS5$_{S}$

E= -3706.91152011 h.

6	1.654457	3.855743	-1.412622

6	2.519783	3.733401	-0.326689
6	2.825420	2.480922	0.196752
6	2.272377	1.318927	-0.363880
6	1.407288	1.451220	-1.461550
6	1.101120	2.706741	-1.976163
6	2.590535	-0.000645	0.190053
6	3.819380	-0.371924	0.887142
8	4.644029	0.198417	1.552869
6	2.263313	-1.350994	-0.366250
16	3.779470	-2.136486	0.316344
16	0.955573	-1.162021	0.808275
16	-0.609478	-3.094103	0.551150
16	-1.518116	-2.742489	-1.262855
16	-3.109199	-1.424291	-1.008611
16	-2.389535	0.510665	-1.264701
16	-1.877102	1.228690	0.648372
16	-3.494867	2.152251	1.390773
9	-4.328851	0.944232	2.146114
1	3.499584	2.393737	1.042588
1	0.965759	0.566046	-1.910053
1	2.959613	4.620258	0.120687
1	0.427164	2.785926	-2.824590
1	1.414532	4.834846	-1.815876
1	2.030813	-1.542049	-1.411447

ProdS

E= -1217.96547608 h.

16	1.371456	-1.273904	1.327726
6	0.538550	-0.100277	0.157280
6	1.419394	1.132526	-0.051755
16	2.726777	0.225303	-0.937530
6	1.502459	-1.044522	-0.461121
6	-0.939236	-0.052377	0.018576
6	-1.644171	1.041065	0.530365
6	-3.028831	1.098510	0.401068
6	-3.716683	0.066894	-0.233229
6	-3.014981	-1.025276	-0.741207
6	-1.631162	-1.086768	-0.618384
8	1.296602	2.289381	0.206473
1	-1.110702	1.846092	1.024563
1	-1.092976	-1.940773	-1.018421
1	-3.569418	1.951738	0.798630
1	-3.545485	-1.831107	-1.238638
1	-4.796722	0.113148	-0.332744
1	1.258724	-1.855186	-1.137865

INT1$_N$

E= -3606.960088 h.

C	-2.026483	0.992177	-0.190252
C	-1.673856	3.449312	-0.299337
O	-1.257514	4.505466	-0.432665
C	-2.391934	2.318095	-0.057448
S	0.693107	1.915595	-1.129137
S	-0.538212	0.289640	-0.692045
S	1.721260	2.296751	0.646119
S	3.509941	1.261734	0.599988
S	3.156567	-0.622960	1.427642
S	3.343256	-2.024906	-0.112294
S	1.592551	-2.142674	-1.240360
S	0.100104	-3.112364	-0.228434
H	-3.380331	2.556708	0.275769
C	-3.081803	-0.081262	0.134754
C	-4.358968	0.298806	0.548118
C	-2.760893	-1.433449	0.015776
C	-5.314735	-0.673169	0.843067
H	-4.611417	1.364917	0.642558
C	-3.717141	-2.405833	0.309829
H	-1.754390	-1.733176	-0.310177
C	-4.993874	-2.025937	0.723555
H	-6.321238	-0.373679	1.169494
H	-3.463959	-3.471892	0.215534
H	-5.747645	-2.792124	0.956111

INT1$_O$

E= -3606.949186 h.

C	1.603675	0.826481	-0.315519
C	2.549521	-1.439748	-0.694120
O	2.723347	-2.542498	-0.939691
C	2.603239	-0.127306	-0.335779
S	-0.318428	-1.277473	-1.320145
S	-0.064490	0.703564	-0.718535
S	-0.885236	-2.291414	0.413223
S	-2.950028	-2.326615	0.503572
S	-3.554367	-0.612423	1.532296
S	-4.534353	0.624572	0.160736
S	-3.170168	1.728185	-0.967275
S	-2.318433	3.232790	0.129267
C	4.030113	0.269837	0.086014
C	4.301149	1.579727	0.482500
C	5.051487	-0.679973	0.071782
C	5.593442	1.939801	0.864006
H	3.495864	2.328492	0.492875
C	6.344017	-0.320193	0.454318
H	4.838006	-1.712459	-0.240513
C	6.615165	0.989479	0.850268
H	5.807287	2.972404	1.175933
H	7.149038	-1.069390	0.443391
H	7.634001	1.273604	1.151170
H	1.866206	1.819694	-0.016343

INT2$_N$

E= -3606.958020 h.

C	-1.919998	0.752656	-0.378991
C	-0.984299	2.970204	-0.304948
O	-0.811740	4.131562	-0.185667
C	-2.054980	2.052680	-0.054120
S	1.729139	2.009914	0.851392

S 0.584720 1.916027 -1.014601
S 0.912926 0.616317 2.081334
S 2.227468 -1.008073 2.142245
S 1.836177 -2.303315 0.543127
S 3.250621 -1.911543 -0.924370
S 2.636660 -0.384378 -2.160169
S -0.412413 0.139483 -1.098395
H -2.965232 2.452546 0.379281
C -3.002062 -0.328274 -0.199195
C -4.243549 0.010405 0.339765
C -2.741046 -1.645849 -0.575246
C -5.223503 -0.968467 0.503227
H -4.448748 1.049094 0.636850
C -3.721536 -2.624947 -0.412713
H -1.762677 -1.912921 -1.000211
C -4.962583 -2.286516 0.126550
H -6.201861 -0.701715 0.928616
H -3.515596 -3.663634 -0.709683
H -5.735417 -3.058138 0.255475

$INT2_O$
E= -3606.950624 h.
C 1.307186 -0.014167 -1.404884
C 1.763157 -1.347712 0.547622
O 2.274734 -1.910193 1.450208
C 2.176546 -0.415167 -0.457659
S -0.841822 -0.485456 1.995236
S -0.206252 -1.709448 0.288212
S -0.763499 1.442707 1.365058
S -2.730211 2.073145 1.041073
S -3.338826 1.547447 -0.893137
S -4.492842 -0.169727 -0.728077
S -3.319348 -1.860349 -0.761077
S -0.370781 -0.606259 -1.418630
C 3.638026 0.068807 -0.419619
C 4.495350 -0.379598 0.585568
C 4.104690 0.955333 -1.390105
C 5.818840 0.059004 0.620574
H 4.126925 -1.077915 1.350981
C 5.428825 1.393395 -1.355803
H 3.429168 1.308707 -2.182524
C 6.285872 0.945511 -0.350612
H 6.494480 -0.293900 1.413206
H 5.796603 2.092063 -2.121360
H 7.329452 1.291149 -0.322841
H 1.531866 0.656048 -2.208146

$TS1_N$
E= -3606.903931 h.
C 1.134981 -2.156074 1.980197
C 2.293176 -0.918310 0.911283
C 2.428807 -2.116605 1.553430
S 0.234710 0.314268 -1.257661
S 0.285864 0.776279 0.766142
S -0.634825 -1.564533 -1.352437
S -2.707508 -1.436542 -1.528729
S -3.398292 -1.206805 0.410653
S -4.144358 0.774099 0.521184
S -2.758637 1.999843 -0.390302
S -1.171346 2.445178 0.835119
O 0.049721 -2.208315 2.366823
H 3.223462 -2.834664 1.751937
C 3.495903 -0.220287 0.249610
C 3.812928 -0.489935 -1.082027
C 4.268246 0.681575 0.981511
C 4.902473 0.141673 -1.681346
H 3.204293 -1.201577 -1.658544
C 5.357564 1.314205 0.381865
H 4.018415 0.894350 2.031006
C 5.674858 1.044313 -0.949308
H 5.152795 -0.071290 -2.730766
H 5.966095 2.025547 0.959061
H 6.533843 1.542235 -1.422080

$TS1_O$
E= -3606.896143 h.
C -1.938195 0.017395 -1.088802
C -1.966012 1.107194 0.592750
C -2.863524 0.415683 -0.170757
S 0.906830 0.452603 1.928885
S 0.588748 1.634119 0.251180
S 0.662474 -1.494414 1.262267
S 2.463345 -2.301932 0.592752
S 2.650699 -1.647949 -1.363870
S 4.260897 -0.269549 -1.354512
S 4.001776 0.960449 0.280554
S 2.626868 2.448010 -0.060416
O -1.179288 -0.321573 -1.888275
H -2.277752 1.674656 1.444633
C -4.378893 0.141351 -0.169885
C -4.882471 -0.990553 0.471733
C -5.247983 1.024495 -0.810423
C -6.254780 -1.239533 0.472186
H -4.196962 -1.687017 0.975971
C -6.620830 0.776103 -0.809218
H -4.851271 1.916753 -1.316015
C -7.124315 -0.355758 -0.168179
H -6.651720 -2.132021 0.977359
H -7.305868 1.472816 -1.313983
H -8.206298 -0.552248 -0.167640

$TS2_N$
E= -3606.953078 h.
C -1.663569 1.023621 -0.389825
C -0.740227 3.284286 -0.077875
O -0.289209 4.334830 0.059895
C -1.710968 2.306986 0.078366
S 2.426348 1.692966 0.095875
S 0.904423 1.915751 -1.361396
S 1.487524 1.072428 1.824953

S 2.073073 -0.902302 2.155826
S 0.925481 -2.195472 0.976042
S 2.150047 -2.874361 -0.573778
S 2.251585 -1.549688 -2.140914
S -0.349536 0.292346 -1.272937
H -2.569999 2.660635 0.646575
C -2.818591 0.042889 -0.114708
C -3.440472 -0.617377 -1.174794
C -3.242957 -0.185402 1.194240
C -4.486924 -1.505205 -0.925952
H -3.106186 -0.436597 -2.206692
C -4.289060 -1.074217 1.443410
H -2.752810 0.334772 2.029886
C -4.911154 -1.734001 0.383589
H -4.977562 -2.025195 -1.761524
H -4.623168 -1.254328 2.475595
H -5.736225 -2.434111 0.579552

TS2$_O$
E= -3606.943562 h.
C -1.153099 -0.392908 -1.298967
C -2.000094 1.586825 -0.105764
O -2.376065 2.521271 0.452355
C -2.115468 0.307259 -0.626592
S 1.312968 1.959554 1.108476
S 0.363241 2.009368 -0.784950
S 0.655450 0.241445 2.042254
S 2.288213 -1.052789 2.146892
S 2.531741 -2.045526 0.320334
S 4.198801 -1.212310 -0.622565
S 3.753198 0.519645 -1.633879
S 0.471201 0.144416 -1.636996
H -1.343301 -1.386524 -1.647464
C -3.500906 -0.312128 -0.364826
C -3.784917 -1.601586 -0.815500
C -4.471767 0.415898 0.322891
C -5.039759 -2.162556 -0.579055
H -3.019552 -2.174995 -1.358325
C -5.726711 -0.145472 0.560352
H -4.248030 1.432137 0.678318
C -6.010885 -1.434436 0.109401
H -5.263904 -3.178677 -0.934799
H -6.491858 0.428624 1.102973
H -7.000192 -1.876971 0.295763

TS3$_N$
E= -3606.951396 h.
C -2.318674 0.741678 -0.021519
C -1.296434 2.434317 1.421658
O -0.740529 3.283226 1.948549
C -2.187138 1.395994 1.174397
S 1.785050 1.352152 -0.837105
S -0.115939 2.394417 -1.078306
S 1.620035 0.556715 1.093790
S 0.931333 -1.357774 0.925101
S 2.644567 -2.501276 0.192773
S 4.288641 -1.317854 0.542035
S 4.425503 0.054534 -0.992073
S -1.502198 0.919487 -1.505938
H -2.741831 1.123483 2.047870
C -3.405464 -0.347114 -0.092371
C -4.612199 -0.173319 0.585904
C -3.183474 -1.506949 -0.834699
C -5.596399 -1.159490 0.522361
H -4.786600 0.740842 1.171682
C -4.168261 -2.493077 -0.899169
H -2.232506 -1.644010 -1.369493
C -5.374544 -2.319605 -0.220695
H -6.547338 -1.022855 1.057470
H -3.993114 -3.407268 -1.484875
H -6.150721 -3.097001 -0.270698

TS3$_O$
E= -3606.941368 h.
C 1.889261 1.078947 1.402306
C 1.925346 1.238761 -1.027312
O 1.862725 1.414210 -2.156097
C 2.399254 0.739031 0.184673
S -1.903060 1.535346 -0.357691
S -0.103550 2.708439 -0.015548
S -1.148756 -0.321655 -0.961693
S -1.005047 -1.513925 0.692155
S -3.066158 -2.040813 1.173094
S -4.148346 -1.691435 -0.542050
S -4.614888 0.317238 -0.606588
S 0.602330 2.119699 1.837387
H 2.370231 0.646140 2.279106
C 3.568720 -0.244025 -0.009112
C 4.028859 -0.536165 -1.293401
C 4.168004 -0.842265 1.099271
C 5.087608 -1.426874 -1.469212
H 3.555679 -0.064948 -2.167069
C 5.227732 -1.732512 0.923637
H 3.805584 -0.611990 2.111573
C 5.687504 -2.024994 -0.360342
H 5.449939 -1.657672 -2.481509
H 5.700316 -2.203732 1.797759
H 6.522313 -2.727209 -0.499207

^{3}INT1$_O$
E= -3606.916656 h.
C 2.082021 2.715925 0.696769
C 1.494700 2.269250 -0.771061
C 2.652546 1.583787 0.150092
S 0.262315 -0.219458 -1.721735
S 0.088734 1.739717 -0.961063
S 0.464228 -1.499264 -0.083208
S -1.230491 -2.682171 0.031317
S -2.628157 -1.715329 1.247926
S -4.165366 -1.084798 0.028600

S -3.662479 0.854491 -0.688266
S -3.619678 2.125895 0.803963
O 1.917906 3.516782 1.595375
H 2.132129 2.935865 -1.633663
C 3.523502 0.491984 0.178630
C 3.959177 -0.034430 1.435510
C 3.883233 -0.200987 -1.009469
C 4.691953 -1.209108 1.478844
H 3.679953 0.480270 2.345743
C 4.626873 -1.364029 -0.948053
H 3.557779 0.194882 -1.965475
C 5.025329 -1.877435 0.296665
H 5.000929 -1.617149 2.436156
H 4.898237 -1.889593 -1.854891
H 5.605994 -2.793585 0.341028

^{3}INT1$_{O}$
E= -3606.940585 h.
C 1.447024 0.621823 -0.492945
C 2.516570 -1.569598 -0.681990
O 2.727891 -2.676601 -0.863199
C 2.528110 -0.222806 -0.392952
S -0.386473 -1.605877 -1.354170
S -0.186796 0.455251 -0.930186
S -1.008999 -2.417436 0.460997
S -3.050743 -2.150581 0.643306
S -3.312262 -0.321285 1.635213
S -4.338318 0.976753 0.365516
S -3.078796 1.825815 -1.068873
S -1.744137 3.137287 -0.243684
C 3.871596 0.232788 0.107094
C 3.964589 0.786680 1.386504
C 5.003218 0.108472 -0.701520
C 5.196838 1.239567 1.845133
H 3.079282 0.861014 2.010393
C 6.233949 0.551530 -0.226585
H 4.921249 -0.315295 -1.698355
C 6.330117 1.118329 1.042341
H 5.272713 1.678394 2.834613
H 7.115198 0.460204 -0.852929
H 7.291178 1.465588 1.408091
H 1.649554 1.663350 -0.236586

^{3}INT2$_{O}$
E= -3606.941463 h.
C -1.800460 1.162943 -0.873296
C -1.792710 -0.093980 1.170082
C -2.472711 0.442153 0.064288
S 0.683572 -1.632689 1.828762
S -0.136501 0.279241 1.509918
S 0.819134 -2.525995 -0.041299
S 2.775095 -2.402968 -0.715397
S 3.020954 -0.567941 -1.679040
S 4.297352 0.558714 -0.497565
S 3.160699 1.598573 0.944064
S 2.195246 3.112781 0.090959
O -1.261502 1.783783 -1.685473
H -2.330858 -0.753949 1.844212
C -3.939553 0.270230 -0.146441
C -4.462435 0.071967 -1.428946
C -4.798536 0.280970 0.957748
C -5.829280 -0.117224 -1.601796
H -3.803350 0.042241 -2.292566
C -6.161658 0.070205 0.778552
H -4.402940 0.457760 1.952946
C -6.680782 -0.126924 -0.499053
H -6.225922 -0.271654 -2.600018
H -6.819925 0.066740 1.641761
H -7.745063 -0.288910 -0.635819

^{3}TS1$_{O}$
E= -3606.900964 h.
C -2.684312 -1.444673 2.012344
C -3.028821 -1.951844 0.698921
C -2.745459 -0.597405 0.880573
S -0.693418 -1.956604 -1.744733
S -1.100251 -3.001908 -0.073270
S 0.655157 -0.128055 -1.132495
S 2.581749 -0.199701 -1.592350
S 3.601296 -1.192720 -0.061720
S 3.363644 0.087274 1.478605
S 2.252898 1.823810 1.185192
S 0.668279 2.006431 0.105786
O -2.508383 -1.639046 3.195574
H -3.719407 -2.561186 0.126587
C -2.698450 0.678889 0.235199
C -2.217582 1.801374 0.933388
C -3.092675 0.802161 -1.110347
C -2.127315 3.025697 0.288353
H -1.914541 1.694979 1.970217
C -2.995379 2.030923 -1.746985
H -3.471577 -0.067523 -1.638383
C -2.512034 3.139481 -1.049570
H -1.753979 3.893521 0.821938
H -3.294987 2.129387 -2.785136
H -2.435966 4.099140 -1.551305

6. References

1. Gaussian 16, Revision A.03, Frisch, M. J.; Trucks, G. W.; Schlegel, H. B.; Scuseria, G. E.; Robb, M. A.; Cheeseman, J. R.; Scalmani, G.; Barone, V.; Petersson, G. A.; Nakatsuji, H.; Li, X.; Caricato, M.; Marenich, A. V.; Bloino, J.; Janesko, B. G.; Gomperts, R.; Mennucci, B.; Hratchian, H. P.; Ortiz, J. V.; Izmaylov, A. F.; Sonnenberg, J. L.; Williams-Young, D.; Ding, F.; Lipparini, F.; Egidi, F.; Goings, J.; Peng, B.; Petrone, A.; Henderson, T.; Ranasinghe, D.; Zakrzewski, V. G.; Gao, J.; Rega, N.; Zheng, G.; Liang, W.; Hada, M.; Ehara, M.; Toyota, K.; Fukuda, R.; Hasegawa, J.; Ishida, M.; Nakajima, T.; Honda, Y.; Kitao, O.; Nakai, H.; Vreven, T.; Throssell, K.; Montgomery, J. A., Jr.; Peralta, J. E.; Ogliaro, F.; Bearpark, M. J.; Heyd, J. J.; Brothers, E. N.; Kudin, K. N.; Staroverov, V. N.; Keith, T. A.; Kobayashi, R.; Normand, J.; Raghavachari, K.; Rendell, A. P.; Burant, J. C.; Iyengar, S. S.; Tomasi, J.; Cossi, M.; Millam, J. M.; Klene, M.; Adamo, C.; Cammi, R.; Ochterski, J. W.; Martin, R. L.; Morokuma, K.; Farkas, O.; Foresman, J. B.; Fox, D. J. Gaussian, Inc., Wallingford CT, 2016.

2. Chai, J.D.; Head-Gordon, M. Long-range corrected hybrid density functionals with damped atom-atom dispersion corrections. *Phys. Chem. Chem. Phys.* **2008**, *10*, 6615-6620.

3. (a) Ditchfield, R., Hehre, W. J., Pople, J. A. Self-Consistent Molecular-Orbital Methods. IX. An Extended Gaussian-Type Basis for Molecular-Orbital Studies of Organic Molecules. *J. Chem. Phys.* **1971,** *54*, 724-728. (b) Hehre, W. J., Ditchfield, R., Pople, J. A. Self-Consistent Molecular Orbital Methods. XII. Further Extensions of Gaussian-Type Basis Sets for Use in Molecular Orbital Studies of Organic Molecules. *J. Chem. Phys.* **1972**, *56*, 2257-2261. (c) Hariharan, P. C., Pople, J. A. The influence of polarization functions on molecular orbital hydrogenation energies. *Theor. Chim. Acta* **1973**, *28*, 213-222. (d) Gordon, M.S. The isomers of silacyclopropane. *Chem. Phys. Lett.* **1980**, *76*, 163-168. (e) Francl, Michelle M., Pietro, William J., Hehre, Warren J., Binkley, J. Stephen, Gordon, Mark S., DeFrees, Douglas J., Pople, John A. Self-consistent molecular orbital methods. XXIII. A polarization-type basis set for second-row elements. *J. Chem. Phys.* **1982**, *77*, 3654-3665.

4. Marenich, A.V.; Cramer, C.J.; Truhlar, D.G. Universal Solvation Model Based on Solute Electron Density and on a Continuum Model of the Solvent Defined by the Bulk Dielectric Constant and Atomic Surface Tensions. *J. Phys. Chem. B,* **2009***, 113,* 6378-6396.

5. a) Dunning, T.H. Gaussian basis sets for use in correlated molecular calculations. I. The atoms boron through neon and hydrogen. *J. Chem. Phys.* **1989**, *90*, 1007-1023. b) Dunning, T.H.; Peterson, K.A.; Wilson, A.K. Gaussian basis sets for use in correlated molecular calculations. X. The atoms aluminum through argon revisited. *J. Chem. Phys*. **2001**, *114*, 9244-9253. c) Kendall, R.A., Dunning, T.H., Harrison, R.J. Electron affinities of the first-row atoms revisited. Systematic basis sets and wave funcions. *J. Chem. Phys.* **1992**, *96*, 6796-6806.d) Woon, D.E., Dunning, T.H. Gaussian basis sets for use in correlated molecular calculations. III. The atoms aluminum through argon. *J. Chem. Phys.* **1993**, *98*, 1358-1371.

6. Grimme, S., Supramolecular Binding Thermodynamics by Dispersion-Corrected Density Functional Theory*. Chem. Eur. J.*, **2012**, *18*, 9955-9964.

7. Luchini, G.; Alegre-Requena, J. V.; Funes-Ardoiz, I.; Paton, R. S. GoodVibes: Automated Thermochemistry for Heterogeneous Computational Chemistry Data. *F1000Research*, **2020**, *9*, 291.

8. Pritchard, B.P.; Altarawy, D.; Didier, B.; Gibson, T.D.; Windus T. L. A New Basis Set Exchange: An Open, Up-to-date Resource for the Molecular Sciences Community. *J. Chem. Inf. Model.* **2019**, *59*, 4814-4820,

9. Harvey, J.; Aschi, M.; Schwarz, H.; Koch, W. The singlet and triplet states of phenyl cation. A hybrid approach for locating minimum energy crossing points between non-interacting potential energy surfaces. *Theor. Chem. Acc.* **1998***, 99,* 95–99.

10. Rodríguez-Guerra, J. **2020**. jaimergp/easymecp: v0.3.2 Zenodo. https://doi.org/10.5281/zenodo.4293422

11. Àlvarez-Moreno, M.; de Graaf, C.; Lopez, N.; Maseras, F.; Poblet, J.M.; Bo, C. Managing the computational chemistry big data problem: the ioChem-BD platform. *J. Chem. Inf. Model.* **2015**, *55*, 95-103.

12. Rivero, P.; Ivanova, V.; Barril, X.; Casampere, M.; Casas, J.; Fabriàs, G.; Díaz, Y.; Matheu, M.I. Targeting dihydroceramide desaturase 1 (Des1): Syntheses of ceramide analogues with a rigid scaffold, inhibitory assays, and AlphaFold2-assisted structural insights reveal cyclopropenone PR280 as a potent inhibitor. *Biorg. Chem.* **2024**, *145*, 107233-107249.

13. a) Wu, J.; Gao, W.-X.; Huang, X.-B.; Zhou, Y.-B.; Liu, M.-C.; Wu, H.-Y. Selective [3 + 2] Cycloaddition of Cyclopropenone Derivatives and Elemental Chalcogens, *Org. Lett.* **2020**, *22*, 5555−5560; b) Brown, R.F.C.; Rae, I.D.; Sternhell, S. *Aust. J. Chem.,* **1965**, *18***,** 1211-19.

14. García, J. J. S., Joo-Cisneros, R. S., García-Bassoco, D., Flores-Alamo, M., Stivalet, J. M. M., García-Valdés, J., & Klimova, E. I. Synthesis, characterization, and oxidation electrochemistry of some novel 1,2-dithiol-3-ones and 1,2-dithiol-3-thiones containing aryl and metallocenyl fragments. *J. Organomet. Chem.* **2021**, *944*, 121809.
